# Software variability in service robotics

**DOI:** 10.1007/s10664-022-10231-5

**Published:** 2022-12-24

**Authors:** Sergio García, Daniel Strüber, Davide Brugali, Alessandro Di Fava, Patrizio Pelliccione, Thorsten Berger

**Affiliations:** 1 University of Gothenburg | Chalmers, Gothenburg, Sweden; 2 Radboud University Nijmegen, Nijmegen, Netherlands; 3 University of Bergamo, Bergamo, Italy; 4 PAL Robotics, Barcelona, Spain; 5 Gran Sasso Science Institute (GSSI), L’Aquila, Italy; 6 Ruhr University Bochum, Bochum, Germany

**Keywords:** Autonomous and (self-)adaptive systems, Service robots, Variability, Robotics software engineering

## Abstract

Robots artificially replicate human capabilities thanks to their software, the main embodiment of intelligence. However, engineering robotics software has become increasingly challenging. Developers need expertise from different disciplines as well as they are faced with heterogeneous hardware and uncertain operating environments. To this end, the software needs to be variable—to customize robots for different customers, hardware, and operating environments. However, variability adds substantial complexity and needs to be managed—yet, ad hoc practices prevail in the robotics domain, challenging effective software reuse, maintenance, and evolution. To improve the situation, we need to enhance our empirical understanding of variability in robotics. We present a multiple-case study on software variability in the vibrant and challenging domain of service robotics. We investigated drivers, practices, methods, and challenges of variability from industrial companies building service robots. We analyzed the state-of-the-practice and the state-of-the-art—the former via an experience report and eleven interviews with two service robotics companies; the latter via a systematic literature review. We triangulated from these sources, reporting observations with actionable recommendations for researchers, tool providers, and practitioners. We formulated hypotheses trying to explain our observations, and also compared the state-of-the-art from the literature with the-state-of-the-practice we observed in our cases. We learned that the level of abstraction in robotics software needs to be raised for simplifying variability management and software integration, while keeping a sufficient level of customization to boost efficiency and effectiveness in their robots’ operation. Planning and realizing variability for specific requirements and implementing robust abstractions permit robotic applications to operate robustly in dynamic environments, which are often only partially known and controllable. With this aim, our companies use a number of mechanisms, some of them based on formalisms used to specify robotic behavior, such as finite-state machines and behavior trees. To foster software reuse, the service robotics domain will greatly benefit from having software components—completely decoupled from hardware—with harmonized and standardized interfaces, and organized in an ecosystem shared among various companies.

## Introduction

Robots are increasingly involved in our everyday life. In contrast to automatized and reprogrammable manipulators—*industrial robots*^1^ used in assembly lines, for instance—*service robots* (https://www.iso.org/standard/55890.html) are autonomous robots that assist human beings by performing useful tasks. The service robotics market is booming worldwide, heading towards a value of 24 billion US dollars by 2022.^2^ Moreover, robots demonstrated being powerful allies of humanity in the fight against COVID-19, the virus that shook the world in 2020. Especially relevant are: (i) disinfecting robots that kill bacteria and viruses in human-populated areas,^3^ as well as (ii) delivery robots that transport items in hospitals,^4^ supporting the staff and allowing safety distancing—both subjects in our paper.

Robots are cyber-physical systems blending hardware and software to interact with their environment. Developing, integrating, and customizing hardware, software, and environmental components adds substantial complexity to robotic systems. Managing this complexity calls for systematic engineering practices as they have been applied successfully to other cyber-physical domains, such as automotive or aeronautics systems. In fact, there is growing pressure on the robotics community to promote well-defined engineering practices that stimulate component supply chains (Bozhinoski et al. 2019), maturing the robotics market. Unfortunately, software engineering (i) has been traditionally considered an auxiliary concern (Brugali and Prassler 2009) and (ii) is still not mature in the robotics domain (García et al. 2020), as witnessed by the absence of best practices in robotics software engineering. This challenges quality assurance, validation, integration, and the autonomy of robotics software.

A core challenge is *variability*—the ability of software to be changed, customized, or configured (Bosch 2004). Robotics software needs to account for a diversity of hardware, operating environments, and customer demands. Similar to other domains faced with variability, such as automotive, avionics, telecommunication, and industrial automation (Berger et al. 2020), the “drivers of variability” are hardware diversity, environment uncertainty, and the different purposes and functions of robots. However, while the drivers and the realization of variability are reasonably well understood in other domains, that is not the case for autonomous robots.

**Fig. 1:**
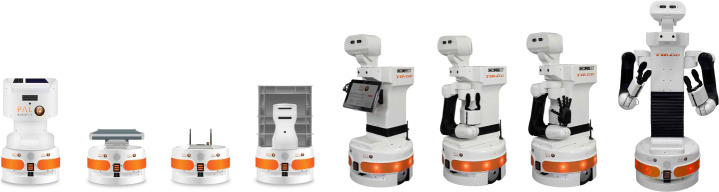
An excerpt of the TIAGo robot family

Consider a robot needing to operate robustly in open-ended environments. To this end, it is typically equipped with a mix of perception, control, planning, learning, and interaction capabilities. The latter depend strongly on the robot’s mechanical structure (e.g., a rover with zero or multiple arms), the missions to be performed (e.g., cleaning a floor, rescuing people after a disaster), and the environmental conditions (e.g., indoor, outdoor, underground). For instance, the robot TIAGo^5^—one of our target robots—is available in many different variants, some of which are illustrated in Fig. 1. Not only the different hardware and mechanical structure but also the missions that TIAGo performs require appropriate mechanisms to deal with variability.

Not properly handled variability easily leads to failures, for instance, through feature interactions (Calder et al. 2003; Apel et al. 2014). Consider a robot with the capability of helping humans transport heavy equipment.^6^ In some variants it also has the feature to simultaneously navigate through the environment, and in many other variants it also has the feature to perform collision avoidance. These features might work well in isolation, but when combined in a variant, a braking command issued by the collision avoidance feature might be overridden by the transportation algorithm commanding the robot to maintain the same path as that of the human.

Our long-term goal is to improve variability management in robotics, where, as we will show, variability is affected by drivers not seen in other domains and is managed in ways lagging behind the state-of-the-art. However, we first need to improve our empirical understanding of variability in service robots—the aim of this study.

We present a study on variability management in service robotics. We investigate the drivers, practices, and challenges of variability. We triangulate from three different data sources: our experiences systematically synthesized in an experience report, an in-depth examination of two companies—i.e., a multiple-case study—based on interviews with nine engineers from two robotics companies, and a systematic literature review (SLR). Our research questions are:

**RQ1:** *What are the drivers of variability in the service robotics domain?* We identify the drivers from our subject companies and describe each driver’s impact on the companies’ practices.
**RQ2:** *What variability management practices are applied by the companies to address the drivers of variability?* We study what practices (i.e., strategies and mechanisms) are applied by our studied companies to manage variability.
**RQ3:** *What challenges do service robotics companies face when managing variability?* We identify the challenges our practitioners face when managing variability for service robots. We discuss their impact on our companies’ development processes.

Our contributions are:

- Qualitative empirical data about variability drivers and realization, together with challenges.
- A literature review on variability management in service robotics.
- A comparison of the respective state-of-the-art and state-of-practice.
- A replication package as an online appendix (García et al. 2021) containing (i) the interview guide, (ii) the codebook from the qualitative analysis, (iii) the literature review protocol, (iv) the used search strings, (v) the data extraction template, and (vi) the detailed literature search results.
- Key observations, proposed hypotheses explaining the phenomena we observed, and actionable recommendations for our intended audience, namely, researchers, tool providers, and practitioners.

**Fig. 2:**
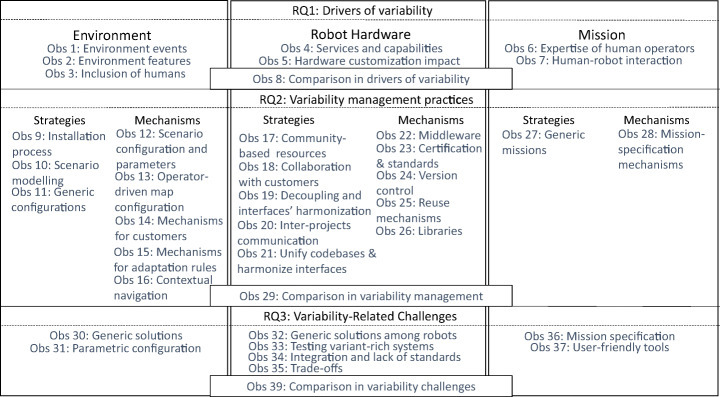
Overview of observations. The figure is structured based on our research questions: in RQ1 we identify three drivers of variability—i.e., environment, robot hardware, and mission—, in RQ2 we list variability-management practices applied by our studied companies, and in RQ3 we discuss challenges related to that management. Each column of the figure represents one of the three drivers of variability identified for RQ1

Figure 2 summarizes our findings. For instance, we learned that (and how) the identified drivers impact development processes, including regulations and standards for safety in open-ended and human-populated environments. Configuration files are a simple, but necessary mechanism to conditionally load software components in robotic applications (e.g., a concrete navigation algorithm). They also parameterize missions the robots perform (e.g., location coordinates in patrolling missions). We also learned that behavior trees (Colledanchise and Ögren 2018; Ghzouli et al. 2020) and finite-state machines (Risler and von Stryk 2008; Dragule et al. 2021a) are mechanisms used by our studied companies to specify both mission and adaptation rules. The most pressing challenge stemming from the identified variability drivers is raising the success rates of configurations and mission specifications that are usable by different robots in various contexts without requiring extensive tuning. We present our findings in detail, throughout the paper highlighting our 38 key observations (labelled as “Obs.” in Fig. 2) with their associated recommendations to researchers and practitioners.

This article significantly extends our previous workshop paper (García et al. 2019b), which only relied on the first of the three data sources: on our experiences (one author has 24 years of experience in robotics) and those of two practitioners of two different organizations developing service robots. We now add two substantial data sources: nine additional interviews, five of them with a company not considered in the previous paper, and a systematic literature review. We also systematically investigate the drivers of variability, the variability-management practices, and the respective challenges. As such, the present article is a multiple-case study based on systematically elicited empirical data from a total of eleven interviews (first and second source) and the literature (third source). The latter allowed us to compare the state-of-practice with the state-of-the-art.

### Organization

Section 2 introduces the required background and terminology for our study. In Section 3 we present the research methodology. In Sections 4, 5, and 6 we describe the results of the study—each section corresponding to one of our research questions, structured along the codebook derived as part of our methodology—and in Section 7 we discuss them. Section 8 lists the potential threats to the study’s validity. In Section 9 we position our study with respect to the related work and conclude in Section 10 with final remarks.

## Background

We now introduce the necessary background on robotics and variability.

### Robotics

Robots are cyber-physical systems embodying a blend of hardware and software that interacts with the environment. The software is typically called a robotic control system, described as a “set of logic control and power functions that allows monitoring and control of the mechanical structure of the robot and communication with the environment (https://www.iso.org/standard/55890.html)”. Although we include findings related to the robots’ hardware, our main focus is on the software of service robots, which “perform useful tasks for humans or equipment excluding industrial automation applications (https://www.iso.org/standard/55890.html)”. These robots differ from industrial robots in that the latter are confined to a well-defined environment and mostly execute a well-defined program to achieve repetitive tasks with high precision. In contrast, service robots often operate in uncertain environments, requiring higher degrees of intelligence and autonomy to handle or transport objects in social or industrial facilities, such as hotels, hospitals, or production plants.

Multiple categories of service robots exist (IFR 2016; IEEE Robots 2020), depending on their scope or application field. The following types of service robots are developed by our robotics companies.

- *Research platforms* are robots with special features suitable for research. They may be used to assess the efficiency of robotics software or newly developed appliances. Therefore, their operation typically requires expert knowledge. Many robots from PAL Robotics belong to this group.
- *Professional service robots* are produced by PAL Robotics and Blue Ocean. Such robots are “used for a commercial task, usually operated by a properly trained operator (https://www.iso.org/standard/55890.html).” They often provide certain services and operate in specific environments, and their main features (e.g., functionalities, hardware, embodiment) are not expected to be changed once released to the market. They typically do not require technical knowledge (e.g., programming skills, robotics engineering) from the operator, although they require some training and an expert to program their behaviors beforehand.

An *operator* is the person designated to start, monitor, and stop the intended operation of a robot (https://www.iso.org/standard/55890.html). She is typically also in charge of commanding tasks and missions (explained shortly). If the operator is not technically skilled, missions are often defined previously by an operator with the required skills:

- *Technical operators* have knowledge of programming languages and are able to use advanced mechanisms for mission specification—e.g., behavior trees (Ghzouli et al. 2020; Colledanchise and Ögren 2018), finite-state machines or programming languages with respective libraries.
- *Non-technical operators* do not possess programming or robotics engineering knowledge and, therefore, typically resort to end-user-oriented, visual environments to specify missions (Dragule et al. 2021a; Ajaykumar et al. 2021).

Another important concept is the *mission*, which expresses the desired behavior of a robot. The implementation of a mission coordinates the robot’s *skills*—programmed actions a robot can perform, often developed as software components by experts—to achieve a mission goal (García et al. 2019a; Ghzouli et al. 2020; Menghi et al. 2018, 2019; Dragule et al. 2021b). An example is: “*A robot* *r*1 *operating in a hospital consisting of a number of rooms and corridors must reach* *room*2 *and disinfect it*.” Note that the terms *mission* and *task* are often used synonymously in the literature, even in reference documents (SPARC 2016). To distinguish both terms, we refer to tasks as repetitive and simpler processes than missions. So, tasks are repetitive and simple coordinated robotic behaviors that are realized as a combination of skills. A mission can be constructed by composing several tasks.

The Robot Operating System (ROS) (Quigley et al. 2009) is the current *de facto* middleware for robotics (García et al. 2020). ROS offers an ecosystem of core software easily extensible by creating or using existing resources in the form of *packages* (Estefo et al. 2019). Packages organize software in ROS and may contain libraries, datasets, configuration files, or third-party software, allowing reuse of robotics software in a standardized packaging format. In ROS, a *node* represents a process that performs specific computational tasks, such as controlling actuators, running navigation algorithms, or processing images.

### Variability Management

Software variability is the ability of a software system to exist in different variants. Among others, variants arise from a diversity of hardware, operating environments, and customer demands—referred to as variability drivers in the remainder. A variety of strategies and mechanisms to manage variability has been proposed (Van der Linden et al. 2007; Apel et al. 2013a; Berger et al. 2014; Nešić et al. 2019; Czarnecki and Eisenecker 2000). In the following, we introduce some of them, ranging from ad hoc to systematic variability management.

*Clone & own* is an ad hoc strategy to create software variants by cloning existing variants and adapting them to the new requirements, changing ownership and decoupling the development lifecycle for the new variants. This strategy is simple and cheap, but does not scale with the number of variants (Dubinsky et al. 2013; Berger et al. 2020; Krueger and Berger 2020; Businge et al. 2022). Clone management frameworks (Rubin et al. 2013; Mahmood et al. 2021) reduce this burden to some extent, but ultimately, organizations often need to re-engineer the cloned variants and integrate them into a configurable platform.

*Configurable platforms* are software systems with variability mechanisms (Apel et al. 2013a; Van der Linden et al. 2007; Berger et al. 2014). These are implementation techniques to realize variation points (places in the source code that differ for individual variants). Since large systems can have many variation points, these are often controlled by *features* (Berger et al. 2015) (explained shortly) modeled in a *feature model* (Berger et al. 2013; Czarnecki et al. 2012; Nešić et al. 2019)—tree-like structures organizing features in a hierarchy, together with constraints among the features. Feature models allow keeping an overview understanding of the platform’s variability and, together with configurator tools (Bashroush et al. 2017; Krueger 2007; Kastner et al. 2009; Hubaux et al. 2012; Franz et al. 2021), allow deriving individual variants in an automated process. Variants are determined by a selection of features—i.e., the *configuration*—that adhere to constraints specified in the feature model.

*Software product line engineering (SPLE)* is the paradigm behind building configurable platforms. it comprises methods, tools, and processes to systematically engineer configurable platforms—i.e., *software product lines*—in a specific application domain (Van der Linden et al. 2007; Apel et al. 2013a; Clements and Northrop 2001; Czarnecki and Eisenecker 2000).

*Features*—distinct and well-understood aspects of a system (Berger et al. 2015)—are an important abstraction to represent the variability of complex configurable platforms. Features are typically developed to be individual and independent units of behavior, but when composed together, may behave differently. This situation, where a feature influences another feature’s behavior, is known as *feature interaction* (Apel et al. 2013b, 2014). To avoid unwanted interactions, developers need to invest time to detect, analyze, and verify interactions, which is especially crucial in safety-critical systems, such as autonomous cars (Juarez Dominguez 2012) or robots (Vierhauser et al. 2019). As such, developers need to manage software variability using proper variability mechanisms. We explore such mechanisms together with related challenges and practices in the remainder.

## Methodology

**Fig. 3:**
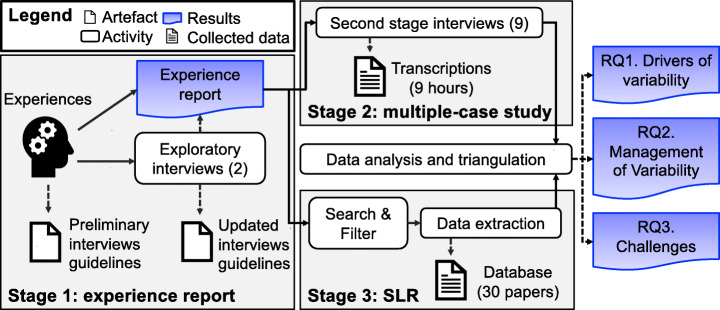
Research methodology overview

We triangulate data and findings from three different sources, which are in the following referred to as stages. In the first stage, we gathered our experiences from different projects and enriched them with two interviews with robotics experts. In the second stage, after identifying the main topics we wanted to explore in detail, we designed a multiple-case study and contacted and interviewed nine robotics experts working for two different companies. The third stage consists of a systematic literature review for which we analyzed 213 papers and selected and extracted data from 30 of them. Figure 3 depicts an overview of our methodology and the stages.

### Stage 1: Experience Report (Authors’ Experiences)

For this stage, we collected our experience on variability management with a focus on service robots. Our experience stems from various EU, academic, and industrial robotics software engineering projects. Bischoff et al. (2010)’s project BRICS aimed to provide researchers and developers with software methods and tools that simplify the configuration of a robot control software system according to the requirements of a given application. A key outcome of BRICS is the HyperFlex toolchain (Gherardi and Brugali 2014), which uses feature models to represent the variation points, variants, and constraints between them using an automated robotic product generation process. The goal of the project Co4Robots^7^ was to develop a framework to support robotic applications to perform complex missions collaboratively (Logothetis et al. 2021; Schillinger et al. 2021). One of the authors has been the coordinator of the IEEE RAS TC on Software Engineering for Robotics (TC-SOFT) for more than ten years. TC-SOFT has promoted yearly workshops and discussion groups with experts in robotics on the synergies between robotics and software engineering, where variability management has been a recurrent topic (Brugali and Prassler 2009). The same author is also an active member of the EuRobotics Topic Group on *Software Engineering, Systems Integration, Systems Engineering*.

In this stage, we conducted two exploratory interviews (see Fig. 3 and Table 1) to collect experiences from the two industrial partners of Co4Robots, namely PAL Robotics and the Bosch Center for Artificial Intelligence (BCAI).^8^ PAL Robotics is involved in several EU projects on, among others, service and industrial robotics, benchmarking robotic frameworks, model-driven methodology, multiple-robot collaboration, and home-assisting robots. The experience at the BCAI stems from a research project on coordinating multiple robots, as detailed by Schillinger et al. (2018). Variability-related challenges in this context primarily concern the governance of different robot configurations being incorporated in a single coordination framework and largely sharing the same software stack.

**Table 1:** List of interviewees. P1 and P2 correspond to our preliminary interviews from Stage 1 and interviewees A–I to those of Stage 2 (see Fig. 3)

	Company	Exp. (years)	Role in the company
P1	PAL Robotics	13	Product Manager
P2	PAL Robotics	10	Software Engineer
I1	PAL Robotics	4	Software Engineer
I2	PAL Robotics	10	Software Engineer
I3	PAL Robotics	14	Software Manager
I4	PAL Robotics	12	Chief Technology Officer
I5	Blue Ocean	16	Senior Robotics Architect
I6	Blue Ocean	5	Robotics Architect
I7	Blue Ocean	7	Senior Robotics Developer
I8	Blue Ocean	3	Senior Robotics Developer
I9	Blue Ocean	4	Senior Robotics Engineer

### Stage 2: Multiple-Case Study (State of Practice)

For the second stage, we obtained rich qualitative data directly from industrial practitioners as opposed to just our own experiences, which, as academic researchers, might be biased. To collect that data, we designed a multiple-case study. Given that our research questions are of an exploratory nature we decided to conduct an *exploratory multiple-case study*, as explained by Easterbrook et al. (2008). Our cases are two companies working on the service robotics domain: PAL Robotics and Blue Ocean Robotics. The chosen cases have multiple embedded *units of analysis* (Easterbrook et al. 2008), since we chose to focus on projects. This decision allowed us to understand the decision-making processes of projects and their interactions with other projects. We planned this stage of our study to be conducted in several iterations where data is collected repeatedly and then analyzed.

#### Selection of Interviewees

The companies that we mainly report on in the first stage of our study work in the areas of robot manufacturing and research. Therefore, both companies primarily work with developers, academics, and researchers, that is, a type of customer knowledgeable in robotics and programming languages. According to our experience in robotics, other companies’ scope is to provide robotic solutions to end-user domains (e.g., hospitals). To increase the comprehensiveness of our study, we included a case of such a type of company in our study, namely Blue Ocean.

We recruited interviewees from PAL Robotics and Blue Ocean following the criteria of heterogeneous roles and experience. We asked interviewees about practitioner colleagues who may fit our selection criteria and might be interested in our study. Following this strategy lead to a variation in the number of interviewees per company, but on the other hand, it gave us a broader number of cases. We did not ask our interviewees about their experience with variability management, we selected instead selected practitioners knowledgeable in robotics and familiar with software development processes.

In total, we conducted nine interviews with nine practitioners from the two companies. Table 1 gives an overview of our interviewees organized by their company and Table 2 gives a short description of each considered company. For every interviewee, we show their experience in robotic in years and their role within the company.

**Table 2:** The two companies considered in our study

**PAL Robotics (Spain)**
Medium-sized robotics manufacturer that mainly produces humanoid robots. One of the authors of the
present article is employed at PAL Robotics. Within the company, each robot platform is assigned to a
product manager. Project teams are not robot-specific but shared among all the business units that
conform PAL Robotics and personnel resources are allocated depending on the work requirements. At
the company, the humanoid robots are considered mainly *research platforms* while TIAGo base^a^ and
StockBot^b^ are considered *professional service robots*.
**Blue Ocean Robotics (Denmark)**
Medium-sized robotics company that offers solutions based on *professional service robots*. The company’s
strategy is to conduct robotics projects that, if succeed on a feasibility exam through validation in the
market, are then constituted as a company, passing to form part of the Blue Ocean’s portfolio. Each
robotics project or company in the portfolio is the project owner of a robot specialized in offering a
specific service. The main areas in which Blue Ocean works are healthcare, hospitality, construction,
and agriculture. Most of Blue Ocean’s customers are end-users from these areas (e.g., hospital staff).

^a^ https://pal-robotics.com/robots/tiago-base/

^b^ https://pal-robotics.com/robots/stockbot/

#### Data Collection

In our study, we used semi-structured interviews to collect qualitative data (Myers and Newman 2007). Semi-structured interviews follow a script prepared beforehand but allow for improvisation, as opposed to structured interviews. This form of data collection allowed us to cover certain question blocks while at the same time the interview could flow freely based on ideas or aspects the interviewee discussed, to which we could choose whether to pay more attention. We were also able to emphasize special topics depending on the interviewee and their role at the company. Concretely, the information provided by practitioners in higher positions of each company’s hierarchy contained more details about organizational aspects of variability management, while software and robotics engineers provided more technical information on development issues, engineering paradigms, and technological spaces.

We designed a semi-structured interview guide and piloted it with our exploratory interviews, whose results were used to establish the final interview guide (which was still slightly refined after each semi-structured interview). We provide the interview guide in our online appendix (García et al. 2021). The interviews ranged from 40 to 76 min, averaging around 60 min. All interviewees agreed to record the interview, amounting to a total of 541 min of interview recordings.

#### Data Analysis

We analyzed the interviews by transcribing them and then performing collaborative iterative open coding (Corbin and Strauss 1990, 2014). In open coding, data is broken down analytically, where *incidents* (i.e., events, actions, or interactions) in the transcribed data are compared with others for similarities and differences. This comparison is a central aspect of open coding, and to accomplish it researchers add conceptual labels that help group together incidents to form categories and subcategories. The result of open coding—i.e., the labeled categories of incidents in the transcribed data—was then documented in our *codebook* (MacQueen et al. 1998). We started creating our codebook by identifying central topics for our study based on our research questions. Based on these central topics we created *a priori* codes, as proposed in the guidelines from Runeson and Höst (2009). Since we read the transcriptions line by line during the analysis we were able to find appropriate codes for specific statements. As suggested by Verner et al. (2009), we used NVivo^9^ to ease the data analysis and document the large amount of collected interview transcript data. Finally, we followed an editing approach, as proposed by Runeson and Höst (2009), for which we created new codes from the firstly defined a priori codes in a hierarchical manner where new interesting topics came up. These codes were continuously revised, which resulted in their occasional merging and splitting.

As suggested by Cornish et al. (2013), performing collaborative coding allowed us to create and refine our codes under different perspectives, which also enriches our understandings and promotes the reliability of our study. The open coding was firstly conducted by one of the researchers to ensure consistency with the use and meanings of the codes. Then, a second researcher was informed of the codes through an informal coding workshop and further refined the coding iteratively. In these iterations, the two authors continuously discussed and refined the codes and organized them in a hierarchy. These iterations led to the definition of 386 codes, 2.6% of them being a priori codes. Many initial codes evolved, and sub-codes were created. For instance, the code “Features acquisition” evolved to three different sub-codes named “How are features identified,” “How are the features modeled,” and “How are features implemented/built.” The two authors in charge of coding then presented and discussed with the rest of the authors the resulting codes to align our knowledge and refine our codebook.

During our data analysis, we strove to establish a *chain of evidence*—as remarked by Verner et al. (2009) and Runeson and Höst (2009)—to provide sufficient information of each step taken in our study so a reader can follow our derivation of results and conclusions from the collected data. Concretely, we discussed the codes between two of the researchers, creating living documents with listings and annotations with that purpose. After some iterations, we created tables, discussed relations, and identified themes, which allowed us to conceive a story to report on the findings for each research question. Some of these artifacts (e.g., our codebook) are provided in our replication package, which can be found in our online appendix (García et al. 2021). Another important artifact is represented in Table 8. It puts together our findings for our research questions based on data from the interviews. The table shows an overview of the main drivers’ characteristics and a mapping to the applied variability management practices and challenges faced by service robotics companies. The mapping was created after several iterations of analyzing the interviews’ data. We also created write-ups that give details on the organizations in which the variability management is done. We append a write-up for each of our studied companies that analyses the collected data based on the BAPO model (Van der Linden et al. 2007) to our replication package.

We provide summaries of our main findings as observations in boxes in the respective sections. For each observation we also provide actionable recommendations for our intended audience, namely researchers, tool providers, and practitioners. Then, the observations are referenced and exploited when we discuss our hypotheses and recommendations.

### Stage 3: Literature Review (State of the Art)

Following established guidelines (Kitchenham and Charters 2007), our systematic literature review comprised three steps: planning the review by defining a search strategy (Section 3.3.1) together with inclusion/exclusion criteria (Section 3.3.2); conducting the review by extracting data from selected papers (Section 3.3.3) and assessing its quality (Section 3.3.4); and documenting the paper selection process (Tables 5, 6 and 7) and the synthesis of results according to our research questions (Sections 4.4, 5.7, and 6.4). Our replication package (García et al. 2021) provides additional artifacts documenting our review, including the review protocol, data extraction templates, and details of our literature selection process.

#### Search Strategy

A first initial search for systematic literature reviews on variability in robotics software (via ACM Digital Library, Scopus, and Google Scholar) by September 2020 revealed that no such publication exists. The search string used for this initial search was divided into three groups of keywords (forming three categories) and applied to the entire content:

1. ("robot" OR "robotic" OR "robotics") AND
2. ("variability" OR "variant") AND
3. ("SLR" OR "literature review" OR "systematic literature review")

After the first search, we understood that Google Scholar is not a digital library but instead a search engine that references multiple digital libraries (Mourão et al. 2020). Google Scholar’s queries also return many unpublished papers and therefore we replaced Google Scholar with IEEE Explore to design a search strategy that balances result quality and review effort. We then defined our search strategy, focusing on the search engines ACM Digital Library, IEEE Explore, and Scopus.

The search query was applied to abstract, title, and keywords without limiting the time range and restricted to the subject areas *Computer science* and *Engineering*. Papers classified only in other subject areas were considered not relevant, such as *Mathematics*, *Materials Science*, *Physics and Astronomy*, *Earth and Planetary Sciences*, *Energy*, and *Decision Sciences*. These papers typically mention robots and software in the context of applications where variability is related to domain-specific aspects, e.g., plant density in wealth crops, leaf nitrogen in coffee, oil spills parameters. Specifically, we created a search string divided into three groups of keywords as follows:

1. ("service robot⋆" OR "autonomous robot⋆" OR "Autonomous guided vehicle⋆" OR "unmanned aerial vehicle⋆") AND
2. ("variability" OR "variant⋆") AND
3. ("software")

With pilot searches, we validated and refined the search string together with the terms and keywords used. For instance, we experimented with adding more terms, such as inspection robot⋆, lawn-mowing robot⋆, vacuuming robot⋆, and entertainment robot⋆, which, however, did not yield more results.

An overview of the search results using both search engines and the string for each filtering iteration is depicted in Table 3. Our first search in Scopus in September 2020 yielded 70 results, from which we filtered out 42 search results using our inclusion/exclusion criteria upon the paper title, keywords, and abstract. We filtered out 10 more papers applying these criteria to the full paper, yielding 18 papers for analysis. We followed the same process for the ACM Digital Library, obtaining 108 search results, then excluding 3 papers and 95, respectively, obtaining a total of 10 papers for analysis. Finally, the search in IEEE Xplore yielded 35 results, from which we filtered out 15 and 18 papers in two steps, resulting in 2 papers.

**Table 3:** Selection process with filtering results

**Scopus**^a^		
First Search^d^	Filtering 1^d^ (− 42)	Filtering 2^f^ (− 10)
70	28	18
**ACM Digital Library**^b^		
First Search	Filtering 1 (− 3)	Filtering 2 (− 95)
108	105	10
**IEEE Xplore**^c^		
First Search	Filtering 1 (-15)	Filtering 2 (− 18)
35	20	2

^a^ Queried Sep. 2020

^b^ Queried Oct. 2020

^c^ Queried Nov. 2021

^d^ Total number of hits using the final search string

^e^ Application of inclusion/exclusion criteria (cf. Table 4) to title, keywords, and abstract

^f^ Application of inclusion/exclusion criteria to entire paper

#### Inclusion and Exclusion Criteria

Table 4 details our inclusion/exclusion criteria. Notably, we did not restrict the scope of our paper selection to a time range. Since, as explained above, no prior literature review on variability in robotics exists, our goal was to obtain a full overview. In fact, our search resulted in papers published between 1989 and 2021, which is a rather large time span for a literature review.

**Table 4:** Inclusion and exclusion criteria

	**Inclusion criteria**
1.	Primary studies.
2.	Studies focusing on service robots.
3.	Studies that relate to robotics software variability.
4.	Studies that identify drivers of variability,
	variability management practices, or variability-related
	challenges.
	**Exclusion criteria**
1.	Studies written in any language other than English.
2.	Short publications and posters (< 3 pages).
3.	Workshop summaries.
4.	Studies focusing on industrial or toy robots.
5.	Studies that do not deal with software variability (e.g.
	mechanical modeling, statistical modeling).

One notable criterion of our exclusion criteria is *Studies focusing on industrial or toy robots*. As specified in Section 1, according to ISO vocabulary, industrial robots and service robots are two different categories of robots. This is particularly true with regards to the driver of variability considered in this paper: the environment of industrial robots is typically structured according to the specific robot work space; the hardware consists of standard manipulator arms, where only the end-effector is replaced; tasks are repetitive and pre-programmed. While entertainment robotics is a subcategory of service robotics considered in our investigation, we conceptualize toy robots as robotic kits consisting of simple mechanical and electronic building blocks.

#### Data Extraction

We extracted data from 30 papers for which we have built a database of the identified drivers of variability, practices, and challenges. The assessment criteria we used to analyze the search results and record the information that we used to answer our research questions is based on the generated codebook from our multiple-case study. Concretely, we created a data extraction template for each digital library (García et al. 2021), whose structures were based on the two top-level codes of our codebook.

The data extraction was performed mainly by one researcher, using the data extraction templates as ground guidelines. The goal was to match our codes from the multiple-case study with the analyzed papers to better triangulate the data between these two sources. We then chose randomly 12 publications out of the total for which a second researcher performed the data extraction independently and the results were compared as a quality assurance check. This triggered a discussion that led to the refinement of our data extraction process. For instance, the researchers agreed to not go deeper than three levels down the codebook’s hierarchy—which is seven levels deep—to keep a balance between the level of detail and complexity. The discussion also allowed the researchers to reach an agreement on fine-grained details, e.g., whether a finite-state-machine-based mechanism for managing mission variability could also be used for environment variability, making the code cross-cutting.

#### Quality Assurance

We assured the quality of our paper selection and analysis as follows. First, defining the inclusion/exclusion criteria that relied on an agreement of five of the authors. Second, while the selection of papers and their analysis were performed by one author, to mitigate potential bias, the results were reviewed and compared by another author—as explained in Section 3.3.3. Once a disagreement was found we involved all the authors, discussed and reached an agreement. In the case of disagreement we would go for majority vote, but it was not needed since we reached the agreement in every case.

The comparison revealed a disagreement on applying the inclusion/exclusion criteria to one paper (i.e., whether the paper is relevant) and five disagreements on the interpretation of data to answer our research questions (i.e., what aspects of our research questions were covered by the search results). In the latter case, the disagreement was concerned with the classification of solutions for the Management of Variability. The selected papers, as well as the majority of the collected papers, do not clearly specify to which driver of variability the proposed solution can be applied. As discussed in Section 5.7, these include the adoption of engineering paradigms such as Model-Driven Engineering, Software Product Line Engineering, Software Frameworks, and Component-Based Software Engineering. One author proposed to exclude these solutions from the data analysis related to Management of Variability, but after a discussion the authors decided to classify them as generic solutions to the Management of Variability for all drivers of variability.

We discussed these disagreements among all authors until consensus was reached. We then also clarified our inclusion/exclusion criteria to minimize selection bias. Third, the same two authors met weekly to discuss the progress of paper selection and analysis. These sanity checks helped us improve our literature review iteratively and maintain its quality by discussing threats to validity and clarifying the methodology.

## Drivers of Variability (RQ1)

Our selection of drivers of variability was strongly influenced by the seminal work by Brooks (1991), who identifies a set of aspects that characterize every robotic system. These aspects were contextualized with variability of robotic systems in the taxonomy of factors by Gherardi (2013). We built upon these studies and triangulated data from the literature, our experiences, and the interviews with practitioners from PAL Robotics and the BCAI to design our list of drivers of variability:

- Environment (based on *robot situatedness* Gherardi 2013). Robots are cyber-physical systems that are situated in the world—instead of being purely software-based agents—which influences the behavior of their systems.
- Robot hardware (based on *robot embodiment* Gherardi 2013). Robots have bodies with which they perceive the external world and operate and manipulate it.
- Mission (based on *robot intelligence* Gherardi 2013). Robots are required to operate based on adequate and useful behaviors, described as missions.

In what follows, we elaborate on the characteristics and impact of the three main drivers— *environment*, *hardware*, and *mission*—on both our studied companies. We highlight the concrete *characteristics* of these drivers identified from our interviews. Thereafter, we report the results from our SLR and contrast them with the findings from the interviews in an observation.

### Environment

Service robots are increasingly expected to work in open environments, often populated by humans, as stated by Bozhinoski et al. (2019) and the H2020 Multi-Annual Robotics Roadmap.^10^ In the taxonomy by Gherardi (2013), this variability driver is related to *robot situatedness*, or *context*—that is, robots operate in a dynamic and complex environment. To this end, robots must be aware of their state and surroundings, which is typically achieved using a variety of sensors as well as navigation and perception algorithms.

Companies need to deal with various characteristics of variability, namely managing (i) different **scenario and map models**, (ii) **events that may occur**, (iii) **specific features of the environment** (e.g., whether humans will populate it), and (iv) **dealing with the inclusion of humans and uncertainty**.

#### Scenario and Map Models

The operation of service robots needs to consider several scenarios, which compile a set of characteristics of an environment and requirements of the robotic application. Engineers (i.e., technical operators) need to model such scenarios and their scope to make their robotic systems able to operate in these contexts. Broader scopes—i.e., those covering more scenarios—lead to more complex modeling. Also, engineers need to consider many details for the modeling of a scenario. For instance, the robot’s maximum speed, which is dictated by its hardware and configuration, will determine execution times, but also how much space it will need to brake if an obstacle is detected. In the case of the farming robot from Blue Ocean, the robot requires a special type of tires to drive on mud, while the motors of other robots from the same company as PTR and UVD must be powerful enough to allow navigation in hospitals (where the maximum slope in corridors is under regulation). Standards also affect the definition of the scenario, e.g., by defining the maximum speed a robot can operate in an environment populated by humans.

For the modeling of scenarios, operators often map the environment to create 3D models, which are used by robots while they operate or by operators to specify regions of interest—as described in Section 5.2.

#### Events

A common characteristic of environment variability highlighted by our interviewees is the modeling of events—phenomena that may occur in the environment where robots operate.^11^ Service robots must be able to cope with such events so to ensure robustness in their operation. Different robot platforms are designed to adapt their behavior based on events from the environment; for instance, any of the studied robots that operate in human-populated environments are expected to avoid collisions with moving objects when navigating. Furthermore, our studied companies need to model adaptation behaviors to specific events that may occur in customer-specific environments, typically handled during the installation process (Obs. 9). This results in these companies managing sets of events for different customers that are later use for mission specification, which makes this topic cross-cutting with the mission driver of variability (see Section 4.3). 
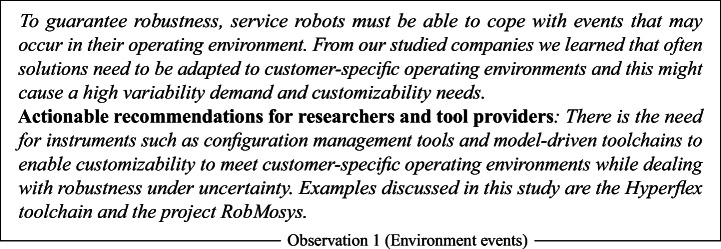


#### Specific Features of the Environment

The environments where robots operate may also pose distinguishing characteristics. From our interviews, we identify a number of distinct categories of environment features:

(i) *Outdoors vs. indoors*. While outdoor environments are inherently challenging due to their proneness to changes (e.g., light and surface conditions), operating indoors presents distinct challenges as well, e.g., robots may be prevented from using GPS or GNSS sensors. Almost all robots of our considered companies (with the exception of Blue Ocean’s farming robot) are dedicated to either outdoor or indoor use.
(ii) *Light conditions*. A well-lit environment rich in visual features requires less powerful components for robot localization (e.g., cameras and localization algorithm) than a poorly-lit or feature-less one. Especially in outdoor environments, the light conditions are prone to change, due to changing weather and daytime.
(iii) *Surface conditions*. Driving on special surfaces (e.g., mud) can make localization challenging because the skidding of wheel tires can make the wheel sensors unreliable. To address this challenge, Blue Ocean uses additional sensors (IMU and GNSS) to support localization. Moreover, stairs are an environment feature posing an insurmountable hurdle for robots that are set up on wheels.
(iv) *Type of obstacles*. The number, size, and dynamic movement of obstacles found in the environment leads to variability in the expectations for the navigation components. A particularly crowded environment may benefit from more sophisticated, adaptive planning components.
(v) *Inclusion of humans.* We dedicated a separate discussion (see next paragraph) to the crucial feature of whether the environment is populated by humans. 
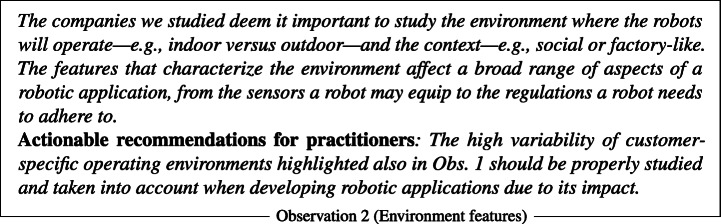

#### Inclusion of Humans

The inclusion of humans in the operating environment imposes several aspects to be considered by the engineers, including safety regulations and uncertainty. Safety regulations may entail, among others constraints, reducing the robot’s speed and, more broadly, maintaining safety instead of reaching a waypoint as the ultimate goal. Based on the inclusion of humans, our studied companies differentiate the environments between “factory-like” or “social.” The former represents factory scenarios (e.g., a storehouse), typically regulated, where efficiency and speed are the aspects to promote. Social environments (e.g., a hospital, a conference) are less structured and normally have an increased presence of humans, who may not behave in a pre-defined or deterministic manner.

The following two examples illustrate two different environment types. A TIAGo base from PAL Robotics is used in an industrial setting (concretely a storehouse) to deliver supplies with the aim of optimizing logistics.^12^ The robot navigates autonomously, but even though it must collaborate with human operators the environment is not highly human-populated. On the other hand, the GoBe, a telepresence robot from GoBe Robots^13^—a project within Blue Ocean’s portfolio—is mainly used to remotely attend to social events such as conferences, hospital visiting, or teaching. I1: *“It’s not the same to be grasping from the top of a table when there’s absolutely no one around than to be grasping on a shelf that is completely filled with stuff, and while other people are roaming around the robot.”* 
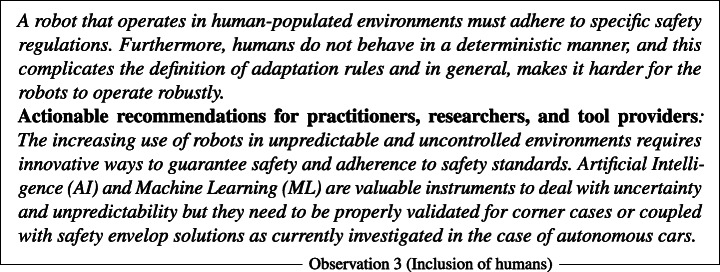


### Robot Hardware

Hardware variability is also a consequence of the cyber-physical nature of robotic systems. Hardware affects the services a robot may provide since they are directly dependent on their capabilities; the locomotion system of a mobile robot allows it to navigate, a robotic manipulator can grasp objects thanks to its robotic arm, and a robot equipped with a camera can “see” the environment where it operates. Due to the cyber-physical nature of robots and the reasons explained in Section 4.1, the environment strongly influences hardware variability. For instance, the context in which robots operate influences their hardware design from the very beginning, e.g., hardware components suitable for an indoor robot may not be adequate for an outdoor robot. Thus, hardware must conform to the requirements of a robot, including the environment where it will operate and the missions it will be commanded to achieve. In this section we describe several characteristics of robot hardware variability, namely (i) **services**, (ii) **robotic capabilities**, (iii) **embodiment**, and (iv) **customer requirements**.

#### Services

Robots are conceived with a purpose, meaning that they are designed to provide specific services. For instance, Blue Ocean’s UVD robot^14^ disinfects hospital rooms and PAL Robotics’ Stockbot helps with retail. The embodiment and hardware design of such robots are tailored to the services they provide. For example, ultraviolet lamps are a specific requirement for disinfecting robots.

#### Robotic Capabilities

To fulfill their expected services, robots must be able to carry on specific capabilities. For example, the PTR robot^15^ was developed to handle patients’ transportation at hospitals. To accomplish this service, the robot needs at least two capabilities, namely to cautiously lift the patient and navigate to the target location. Specific mechanisms and sensors are required for the robots to perform such capabilities. For instance, a robot would need some sort of gripping actuator to grasp objects. Despite efforts from the companies to harmonize solutions and interfaces to ease the management of variability among their robots (see Obs. 19), the set of specific capabilities of each of their robots entail another source of variability. The hardware significantly differs between two robots of the same company as is the case of UVD and PTR robots because their intended services and thus capabilities are different. 
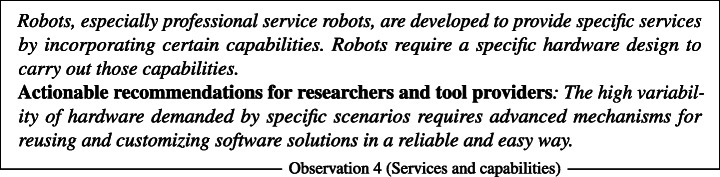


#### Embodiment

One of the most consequential features of a robot is the mechanical *embodiment*, as discussed by Gherardi (2013). Recent studies (Ventre-Dominey et al. 2019) have demonstrated that the embodiment of a service robot can increase social closeness and acceptability by its users. The embodiment can affect the hardware design of a robot due to various factors, including its size, e.g., an RGBD camera might be too big to substitute a monocular camera. Another aspect of the robot influenced by the mechanical side is the design and selection of hardware; for instance, different types of motors and actuators would require different motor controllers, drivers, or feedback sensors. The embodiment directly affects the software since the robotic sensors and actuators describe which capabilities and services a robot can perform. For instance, different navigation algorithms are used based on the kinematics of a robot. That is, a drone would require a different navigation algorithm than a ground robot, and in turn, a differential drive would require a different navigation algorithm than an omnidirectional one.

#### Hardware-Related Customer Requirements

**Fig. 4:**
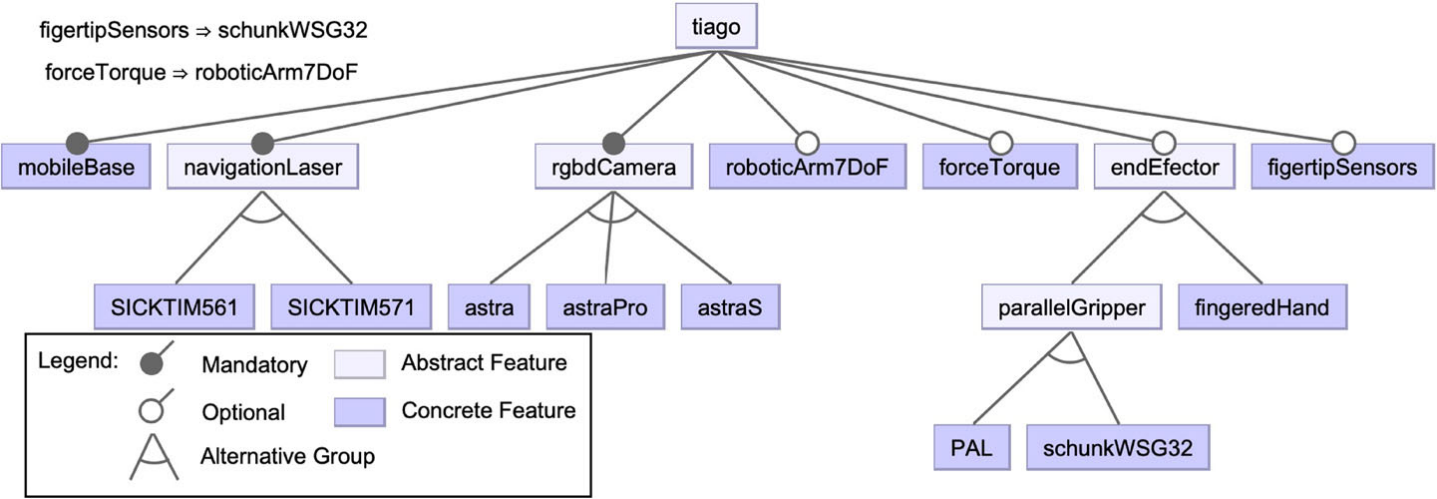
Feature model of the TIAGo robot (excerpt). The model shows several features that a TIAGo robot may incorporate and the versions that can be equipped (e.g., different types of navigation lasers or RGBD cameras). The cross-tree constraints at the top-left indicate that the equippment of fingertip sensors require a specific parallel gripper and the force-torque sensor a robotic arm with seven degrees of freedom (DoF)

As described in Section 3, the scope of both companies is different and it hugely affects the impact of hardware variability in each of them. PAL Robotics mainly manufactures research platforms whose hardware modules can be configured by customers, as described in leaflets of their products, having more than 30 variants for one of them, i.e., TIAGo.^16^ An excerpt of the possible features of this robot is depicted in the feature model of Fig. 4. Differences between the configurations based on customer requirements generally lead to static variability in this context. Hardware choices of sensors and actuators define the required interfaces and controllers to be deployed into de robot. Although PAL Robotics creates variants of their robots based on tailored customer requirements, the company also provides some pre-defined variants of their products, e.g., TIAGo Iron, Steel, Titanium, and TIAGo++ (see Fig. 1). As opposed, Blue Ocean’s robots are considered professional service robots, and thus, once released to the market their hardware design is fixed. 
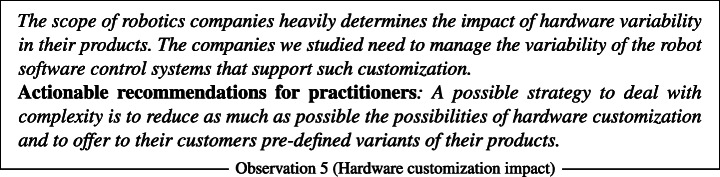

### Mission

We define missions as coordinated combinations of skills that express the desired goals of the robots. Missions must be specified by operators, either customers (e.g., end-users, developers) or engineers at the company. Missions possess several characteristics of drivers of variability, namely (i) **expertise of the human operator**, (ii) **means of human-robot interaction**, and (iii) **expected and unexpected events** (already discussed in Section 4.1). Both companies strive to raise the levels of abstraction of their mission specification methods to promote reusability, modularity, and improve their user-friendliness.

#### Expertise of Human Operator

As described in Section 3, the scope of each of the studied companies varies, resulting in a difference between customer groups. As detailed in Section 5.6 technical operators, since they have knowledge of programming languages, are able to use advanced mechanisms for mission specification—e.g., behavior trees, finite-state machines, general-purpose languages. PAL Robotics’s main portion of customers is developers with programming skills. Operators working with professional service robots from Blue Ocean are mostly non-technical operators and therefore cannot modify the underlying mission used by the robotic application and instead can only modify some parameters via a GUI. A reason for this policy, apart from the expertise it would require, is safety concerns. Allowing non-technical operators without technical and safety regulations knowledge to modify the underlying missions would breach the safety of the robots. To a lesser extent, PAL Robotics also provides robotic applications to non-technical operators, specifically based on TIAGo Base and Stockbot. 
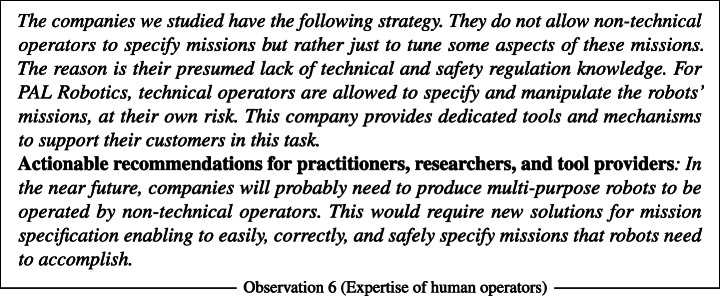


#### Means of Human-Robot Interaction

It includes mechanisms and strategies between robots and operators. The operator needs to communicate the mission to be executed by the robot and the robot might need to communicate when it has completed the mission. The ISO 8373:2012^1^ defines human-robot interaction as the “*information and action exchanges between human and robot to perform a task by means of a user interface*” and in turn, user interface as “*means for information and action exchanges between human and robot during human-robot interaction*.” For instance, robots may communicate the state of the mission they are performing (e.g., changing behavior to charging mode), which could be accomplished by prompting a message in a graphical user interface (as is the case of UVD robots), via voice commands or flashing LEDs. Interaction via communication could also comprehend altering aspects of the mission at runtime (e.g., the operator specifies to a robot which object to grasp^17^), which could be performed using buttons in the robot, a GUI, or gestures (García et al. 2018). 
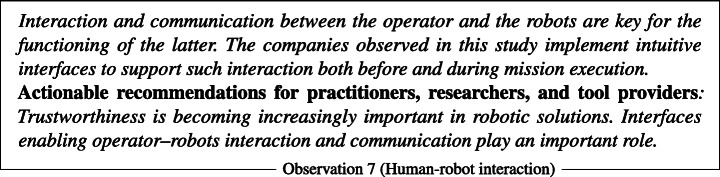


### Drivers of Variability from the Literature

We now present the results from our systematic literature review (SLR) that identifies drivers of variability in robotics. Table 5 provides an overview of the analyzed papers on the topic, indicating with black dots which driver of variability is addressed in each paper. While conducting our SLR, we realized that the selected papers were concerned with two main types of robots. Concretely, 17 papers refer to wheeled ground robots (with or without an onboard robotic arm) used in indoor environments, while 13 papers refer to professional or low-cost unmanned air vehicles (UAV). We will highlight this distinction in every table listing our SLR results.

**Table 5:** Drivers of variability from the literature (RQ1)

			E^a^	H^b^	M^c^
Ground robots	P1	Lee et al. (2006)			∙
	P2	Álvarez et al. (2006)	∙	∙	∙
	P3	Kimour et al. (2009)	∙	∙	∙
	P4	Steck and Schlegel (2011)	∙	∙	∙
	P5	Lotz et al. (2013)	∙	∙	∙
	P6	Brugali and Gherardi (2016)	∙	∙	∙
	P7	Brugali and Valota (2016)	∙	∙	∙
	P8	Brugali and Hochgeschwender (2017)	∙	∙	∙
	P9	Brugali and Hochgeschwender (2018)	∙	∙	∙
	P10	Brugali et al. (2018)	∙	∙	∙
	P11	Rollenhagen et al. (2019)	∙	∙	∙
	P12	Wirkus et al. (2020)			∙
	P13	Seiger et al. (2015)			∙
	P14	Niemczyk and Geihs (2015)	∙	∙	∙
	P15	Goldsby and Cheng (2008)			∙
	P16	Saglietti and Meitner (2016)			∙
	P17	Buchmann et al. (2015)		∙	
UAVs	P18	Brown et al. (2007)	∙	∙	∙
	P19	Steiner et al. (2013)	∙	∙	
	P20	Silva et al. (2013)	∙	∙	∙
	P21	Fragal et al. (2013)	∙	∙	∙
	P22	Ozdemir et al. (2014)	∙	∙	∙
	P23	Queiroz and Braga (2014)	∙		∙
	P24	Czerniejewski et al. (2016)	∙	∙	∙
	P25	Feng et al. (2015)			∙
	P26	Braga et al. (2012)	∙	∙	∙
	P27	Olaechea et al. (2018)	∙	∙	∙
	P28	Brooks and Iagnemma (2009)	∙		
	P29	Pant et al. (2015)	∙	∙	∙

^a^ Environment variability

^b^ Hardware variability

^c^ Mission variability

The drivers of variability are mostly described in the introduction section of each paper and used as motivation for the proposed scientific approach. We interpret this data as an indicator of the relevance of the topic addressed by our investigation.

Most papers (29 out of 30) identify one or more of the three drivers of variability that we hypothesized when we formulated RQ1. We interpret this as a confirmation of the significance of the research question.

Regarding the drivers of variability investigated in our study, environment variability is addressed more explicitly in papers that refer to ground robots as they have to operate in everyday open-ended environments with changing operational conditions (e.g., illumination) that affect the correct acquisition of sensory measurements. Some papers focus on robotic applications working in diverse environments, as the study by Álvarez et al. (2006), which discusses the variety of ship types and shipyards their robotic applications must adapt to. The main horizontal domain to which UAVs of the studied papers are applied is agriculture, where environment variability mostly consists of the different field types during agricultural tasks.

Hardware variability in ground robots typically refers to the variety of sensors that can be used for common robot functionalities, while for UAV robots it refers to differences in the mechanical structure.

Service robots operating in hostile environments, such as nuclear plants, vessel internals, and disaster scenarios (Álvarez et al. 2006; Niemczyk and Geihs 2015), are equipped with specific sensors (e.g., RGB cameras, infrared cameras, depth cameras) according to the task to be performed and the operational conditions (e.g., illumination, radiations).

Home service robots (Kimour et al. 2009) perform tasks that require interaction with humans using a variety of human-robot interfaces, such as physical buttons, microphones, speakers, cameras, and touch screens.

Service robots for logistics and factory automation (Brugali and Valota 2016; Rollenhagen et al. 2019) consist of mobile manipulation platforms that can be customized for the transportation and manipulation of various types of loads. Hardware customization requires adequately configuring kinematics, dynamics, and control parameters (e.g., speed, acceleration, impedance). Parameters configuration might be performed before the execution of a task (i.e., at startup) or even during the execution of a task (i.e., at runtime); for example, when the robot automatically changes the manipulation tool.

Similarly, UAVs used for service robotics tasks have a customizable kinematics structure. This permits, for instance, to dynamically activate or deactivate additional motors for short-distance transportation of heavy loads or long distance transportation of lighter loads (Olaechea et al. 2018; Silva et al. 2013; Fragal et al. 2013) and change the carried tool (e.g., a thermal camera for transmission line inspection or an RGB camera for traffic monitoring) (Braga et al. 2012; Czerniejewski et al. 2016; Brown et al. 2007). Different kinematics structures account for variability in the flight operations, as for example as taking off from limited runway space or using a parachute for landing (Ozdemir et al. 2014; Steiner et al. 2013).

Robot capabilities (e.g., mobility, manipulation, user interaction) are greatly affected by the available hardware resources (i.e., sensors, actuators) as hardware variability induces a corresponding variability in the software implementation of common functionality, such as perception and motion control. This variability demands for architectural design approaches that promote flexibility and configuration of the robot control system.

Mission variability is discussed in relation to the specific purpose and application of the robotic system—e.g., cleaning of ship-hull surfaces (Álvarez et al. 2006), home entertainment (Kimour et al. 2009), and factory logistics (Rollenhagen et al. 2019). A concrete example of mission variability for UAVs consists of different payloads that require fine-tuning of flight control parameters. Two papers (Niemczyk and Geihs 2015; Rollenhagen et al. 2019) mention robotic applications working with humans, as of rescue robots by Niemczyk and Geihs (2015).

In addition, some papers identify new drivers of variability. Concretely, the study by Lotz et al. (2013) identifies variability associated with Quality of Service (QoS), including non-functional properties like safety. In this paper, the authors use the example of a robot running out of battery that might prioritize power consumption over task efficiency to fulfill its mission. 
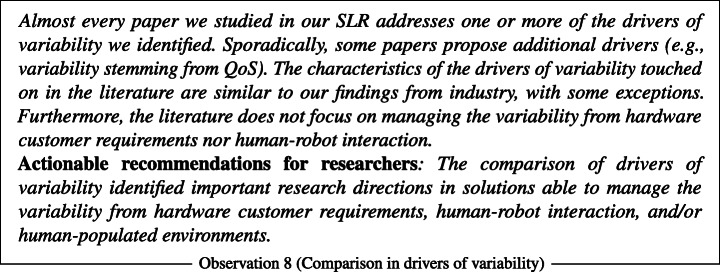


## Variability Management Practices (RQ2)

Each driver of variability entails different challenges and, therefore, requires special strategies and mechanisms to manage them. In our study, strategies refer to more abstract ways of tackling a specific problem (e.g., decoupling services provided by a robot from specific sensors), while mechanisms refer to technical approaches (e.g., using behavior trees to define the robotic missions). The variability drivers studied in this paper are related to each other, thus, some of the practices applied by companies are cross-cutting, intending to address multiple variability drivers. For instance, the adaptation rules used by companies to make their robots adapt to the environment are also used during mission specification to define possible robotic behaviors. For each of the three identified variability drivers, one subsection is devoted to strategies and mechanisms applied for addressing it. A final subsection presents results from our SLR and concludes with an observation of the triangulation of those results with our interviews’ findings.

### Environment: Strategies

The main strategies we identified for environment variability management are (i) **installation process**, (ii) **scenario modeling**, (iii) **generic configurations**, and (iv) collecting and analyzing **customers’ feedback**.

#### Installation Process

It is a well-known term in industry for setting a robot to a new environment. The term has been already standardized in the ISO 8373:2012. During this process, the robots are “installed” in the new environment by mapping the area, setting regions of interest, and creating event-catching solutions. As anticipated in Section 4, the drivers of variability studied in this paper are sometimes intertwined. The installation process also concerns the mission driver since during this process missions can be already defined by a knowledgeable operator from the company.

Our studied companies support customers during the installation process since it cannot be completely automated and requires specialized knowledge. According to two interviewees, Blue Ocean is developing tools that will enable end-user customers to perform the installation. The same company has also performed this process remotely due to the situation caused by COVID19, which still requires the involvement of an expert operator. 
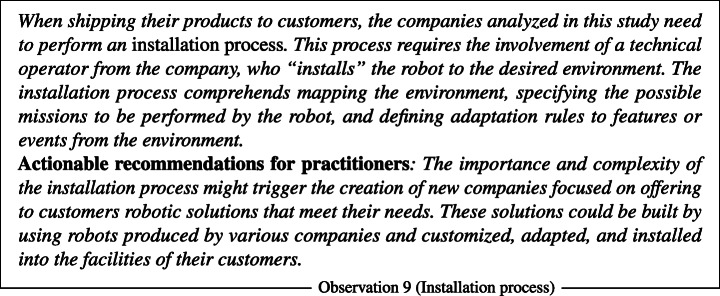


#### Scenario Modeling

It is performed by our studied companies in the first steps to develop a robotic application. As explained in Section 4.1, the modeling of a scenario encompasses identifying the requirements of a robotic application to cover certain scenarios and the characteristics and constraints of the operating environment. 
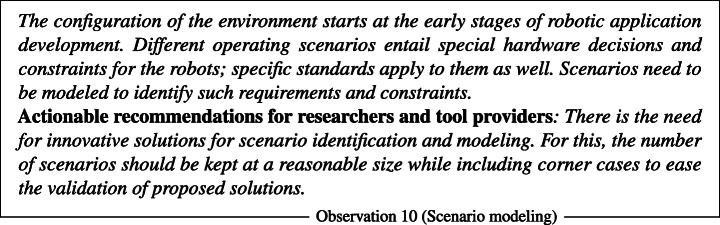


#### Generic Configurations

Besides those early-stage decisions, the studied companies also configure their robotic applications based on the environment using configuration files and parameters. These mechanisms will be further explained in Section 5.2. The purpose of making configurations tailored to a specific environment is to maximize the efficiency of robotic applications in certain scenarios. On the other hand, the studied companies opt to configure their robotic applications by including generic values that are given by rules or features of the environment. The goal is to create generic solutions that work in most of the scenarios without having to fine-tune parameters for each context with the overhead in terms of effort it would entail. For instance, in the case of configurations made for UVD robots, some values are given by the regulations of hospitals—i.e., their most typical operating environment. I9: *“The process of selecting the wheels that we would have to use [...] takes into account the maximum slope that you would find in a hospital, and that’s because there is a maximum slope that someone can climb using a wheelchair.”* In the case of PAL Robotics, the Stockbock robot always works with the same configuration of parameters, which are produced during the installation process. 
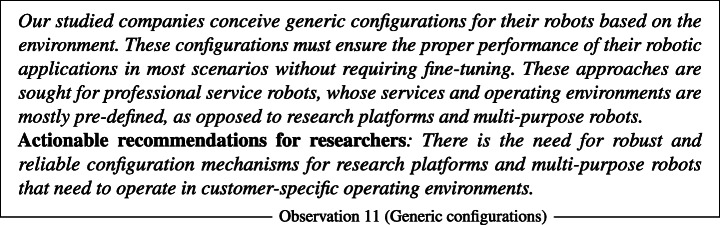


#### Customers’ Feedback

The feedback from customers can be used to update the models and knowledge of the companies of the environments where their robots operate. These updates may lead to the tuning of existent configurations or to identify and model new events that our studied companies use to generate adaptation rules for their robots. I2: *“If we have permission to do so, we collect the data [from a reported error from the customer] and then we can reconstruct what happened. [For instance,] in this situation the laser beam reflected on a mirror, which caused the crashing of the robot’s operation. We can integrate this situation into the set of situations that we tried to handle with a generic set of parameters.”*

### Environment: Mechanisms

In the following, we discuss mechanisms used for making configurations based on the environment broadly divided into operator-driven and self-configuration.

#### Operator-Driven Configuration

Several mechanisms are applied by both companies to manage the variability coming from the environment, mostly to configure the operating scenario and its map (before run-time) and for the robotic application to adapt to such an environment (at run-time). Specifically, in this section we discuss as mechanisms (i) **parameters**, (ii) **configuration files**, (iii) **map-editing tools**, and (iv) **mechanisms for customers**.

##### Parameters

Both companies make use of parameters to define some aspects of robotic applications for specific operating scenarios. These parameters are primarily tuned when launching the required packages to run the scenario but could be changed during run-time as well. Some of those parameters are low-level details of certain algorithms, e.g., the drifting rate of a navigation algorithm, as stated by a PAL Robotics interviewee. Both companies report on the usage of a tool from the ROS ecosystem, namely rqt_reconfigure.^18^ The tool permits tuning parameters both before and during run-time.

This mechanism is cross-cutting with hardware variability (see Section 5.4): to promote the reusability of components and skills among their platforms, PAL Robotics uses parameters to configure their codebase (Obs. 21).

##### Configuration Files

Configuration files are used by both companies to adjust the configuration of their robots. Commonly used configuration files in ROS are yaml^19^ and roslaunch^20^ files. The first use the well-known YAML format to easily loading sets of configuration parameters (e.g., from calibration) and the latter is a tool that allows launching multiple ROS nodes while at the same time setting parameters on the ROS parameter server. Configuration files are used by both companies to launch packages and software components required for specific scenarios as well as for defining values for parameters. According to interviewees from PAL Robotics, configuration files are created in a rather ad hoc way at the company. I1: *“At the end, what we have is a bunch of configuration systems for each different scenario that we’ve been in.”*

Configuration files are also used as a mechanism for hardware variability management (Section 5.4). Concretely, PAL Robotics makes use of configuration files to load sets of parameters to make certain skills usable by different robots. Tuning parameters and loading configuration files are two mechanisms used together at PAL Robotics to keep a unified, common codebase that is also configurable. PAL Robotics implements robot-specific configuration files that are loaded at startup. These are specific YAML files to robots’ serial numbers that specify the dependencies and libraries required for the functioning of each robot (Observations 25, 26). 
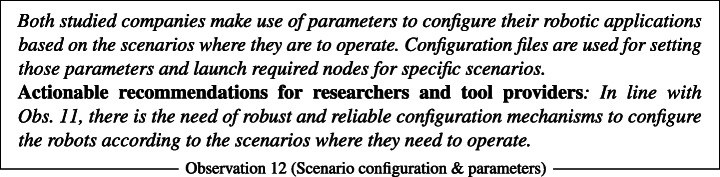


##### Map-editing Tools

Both companies model the environment and provide tools to configure those models. Blue Ocean provides an application to its customers with which they can configure the map of the environment by making map annotations and removing or adding virtual obstacles. It does not require specialized knowledge from the operator, since the typical customer target of the company are non-technical operators—e.g., staff from a hospital. PAL Robotics provides a similar editor on-demand, which is a rviz^21^ plugin. The editor adds functionalities to rviz including downloading and uploading maps, changing the active map, as well as defining virtual obstacles and points and groups of interest. 
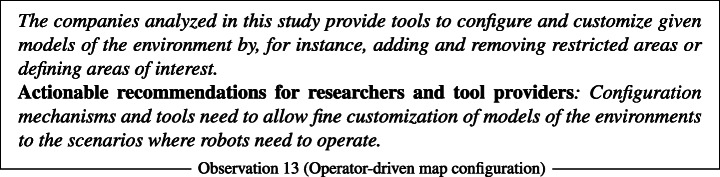


##### Mechanisms for Customers

Generally, configurations based on the environment are to be made either by the company, the system integrator, or self-tuned by the robots, especially if the customer is a non-technical operator. In Blue Ocean, most environment-related configurations are made by an expert practitioner or autonomously by the robots during run-time. However, this company also allows customers without programming knowledge to tune some parameters of the environment model. For instance, the time a UVD robot needs to stay in every position to consider it disinfected is a parameter a customer can tune by using a graphical user interface (GUI) in a tablet. Since PAL Robotics also provides services to researchers and developers, the company provides specific interfaces and an API to allow technical operators to configure their systems. 
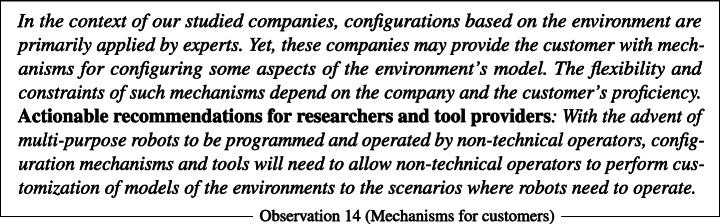


#### Self-configuration

Since service robots typically operate in open environments, they must be able to self-configure or *adapt* to their operating context. As mechanisms for self-configuration we identified (i) **adaptation rules**, (ii) **contextual navigation**, and (iii) **in-house tools**.

##### Adaptation Rules

Both studied companies conceive adaptation rules that are applied based on conditions that are predefined before execution. Adaptation rules may also be used for failure detection, recovering after failing (i.e., fallback behaviors), and to define safety rules that override the running controller. Adaptation rules can be defined using systematic mechanisms (described shortly) or can be also hard-coded into the robot control system. As stated by an interviewee from PAL Robotics, a problem related to the generation of hard-coded rules is that they may grow to a number difficult to manage after some time.

At PAL Robotics, developers use an in-house variant of SMACH^22^ to define finite-state machines (FSMs), which are the mechanism to define the adaptation rules and robotic missions at the company. According to one interviewee from the company, one advantage of using SMACH is that it allows updating the FSMs after compilation time. All PAL Robotics interviewees acknowledge that they are planning to migrate from FSM to behavior trees for generating robotic missions and adaptation rules. The main reason they gave is that behavior trees are easier to understand by humans.

As opposed to PAL Robotics, Blue Ocean currently uses behavior trees to define adaptation rules and their robotic missions; for a better understanding of behavior trees we refer to the work of Colledanchise and Ögren (2018). At Blue Ocean, software components in the lower-level layers of their architectures are used to monitor the environment and trigger events (Obs. 1) that may affect the behavior tree of the robotic application. I9: *“The way that we are following the adaptations now is we have some software bits that are responsible for watching the external events, and then they can trigger another event to have the behavior tree follow another path or reconfigure using other parameters.”*

We elaborate on these mechanisms in Section 5.6. 
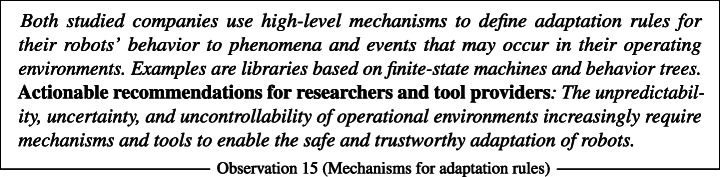


##### Contextual Navigation

In point-to-point navigation, distance or time are the dimensions typically optimized. However, in open environments, other factors like minimum distance to a human due to safety reasons or due to what is considered socially acceptable must be considered. The goal of contextual navigation is to adapt the navigation of the robot to the specific and instantaneous current context where it is operating (Lu 2014). A contextual navigation solution would load or unload planning algorithms or configure them based on the context detected for each instant by the robot. This approach aims to increase the success rate of the algorithm. A concrete example given by a Blue Ocean interviewee is the process of a UVD robot crossing a door; in that situation, the robot will detect a change of context and will need to tune some parameters of its working planner or load a more appropriate planner for the new task. Within Blue Ocean, the UVD project is planning to integrate this approach in a near future. The farm-robot project has already applied this approach to their robotic applications, which are able to load and unload appropriate planners when a change in the context (e.g., indoors or outdoors setting) is detected.

##### In-house Tools

While Blue Ocean partially relies on standard tools, including the ROS middleware, their contextual navigation mechanism (see previous paragraph) is implemented in a custom in-house framework. The framework allows developers to define navigation skills by implementing them with available planners and ROS-provided functionality. Examples of such skills are covering an area as much as possible, following a line, or following a certain talking thing. The framework is responsible for monitoring the context of the robot and for responding to changes of context (e.g., moving through a door, or observing that the current room is getting crowded with people) by exchanging or re-configuring the used navigation skills. 
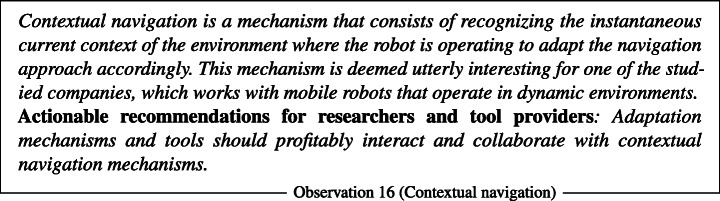


### Hardware: Strategies

Due to the cyber-physical nature of robots, hardware massively impacts many phases of their development. Hardware is typically decided during the design stage of the robot, but at later stages also influences the software skills it may implement. Furthermore, one of our studied companies made robotic hardware configuration a company policy for their robots.

Strategies for managing hardware variability in both studied companies mainly serve two topics, namely developing robotic skills and hardware customization.

#### For the Development of Robotic Skills

This section discusses strategies used by companies to develop robotic skills, namely (i) the **reuse of resources**, (ii) the **collaboration with customers**, (iii) **iterative development and documentation**, (iv) **decoupling**, (v) **harmonized interfaces**, and (vi) **inter-department communication**.

##### Reuse of Resources

Instead of developing skills from scratch, both companies strive to reuse already available software components for specific skills when possible. According to two interviewees, this sometimes requires modifying the components to specific needs, especially when reusing packages from the internet. Examples of such modifications are parameters tuning, as the distance between the robot’s wheels or the position of a specific sensor on the chassis of the robot. An interviewee mentions that licensing constraints impeding the usage of certain resources might exist. 
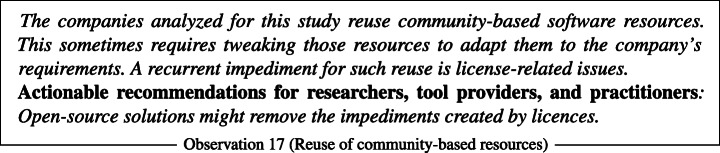


##### Collaboration with Customers

Regarding skills reuse, a factor that greatly impacts each company’s strategy is its target clients—mainly technical operators for PAL Robotics and non-technical operators for Blue Ocean (Obs. 6). A consequence of this is that PAL Robotics can benefit from solutions developed by their clients if they consent. I4: *“One example is the [research group from] Koblenz University, they use the TIAGo for the Robocup@Home competition. And they developed the complete application of the robot, which understands natural language commands and autonomously navigates in a domestic environment, opens a fridge, grasps an object, and brings it back to the initial user. In so they go even beyond what we have been able to do.”* However, according to the CTO from PAL Robotics, integrating such solutions into the company’s system is complicated or sometimes impossible due to the diversity of the used developing tools I4: *“It’s complicated for us to integrate back all this knowledge and all these functionalities because sometimes they use the new version of libraries, a new version of a sensor [...]. So let’s say that because of the real variability of the use case it is really difficult for us to inject back all this functionality.”*

A possible and interesting outcome of this collaboration with customers is the creation of an ecosystem where solutions from both the company and customers must conform to given rules. This ecosystem, similar to ROS’s ecosystem but thoroughly tested and documented so to make it company-complaint would facilitate software reuse. This may reduce the overhead caused by skills and glue code development and provide a new way to manage their associated variability. Supporting practitioners to find specific solutions for their concerns would help to reduce the impact of the “reinvent the wheel” phenomenon discussed by García et al. (2020). Three interviewees from PAL Robotics and one from Blue Ocean find such an ecosystem valuable for the development pace in the robotics domain. 
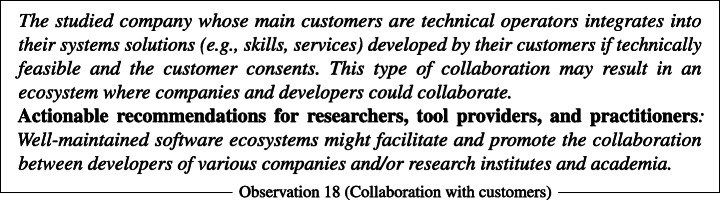


##### Decoupling

Despite the efforts from both companies to reuse existing resources, they also need to develop new skills for their robotic applications. Developers from both companies strive to decouple their software from hardware. The main goal is that hardware can evolve without affecting the codebase. As an example, services are decoupled from specific sensors, so a specific brand of camera is not required for detecting objects as far as the camera provides an image stream. One interviewee from Blue Ocean describes the application of the 5Cs method to decouple computation, communication, configuration, coordination, and composition in their robotic applications.

##### Iterative Development and Documentation

One interviewee from Blue Ocean states that the skills’ development is performed iteratively through a try and error process. Another interviewee from the same company declares that this development process is thoroughly documented, detailing the tasks to perform and the time expectations.

##### Harmonized Interfaces

To simplify the usage and development of robotic skills, both studied companies strive to harmonize the interfaces among their software components: they put effort into explicitly defining their interfaces in a way that bridges the heterogeneity of different software and hardware components. In our interviews, we found three main practices being applied towards this goal: (i) Relying on software development paradigms that encourage developers to explicitly think and reason about interfaces. I6: *“That is because of the service-oriented architecture, which requires to put focus on the contracts, and that is what we do.”* (ii) Decoupling robotics skills from hardware via minimal interfaces. I4: *“If you need to move a robot in a certain environment, you need to define an interface and some resources that are the minimum requested by the algorithm for making this possible. But it’s better to make it decoupled from the hardware because the hardware is going to evolve.”* (iii) Separating the 5C. I1: *“One of the concepts I learned in the past and I promote with my colleagues is the separation of the 5C: [...] computation, communication, configuration, coordination, and composition. This means that, for example, you want to have your component configurable, and nowadays we have many ways to do that, your URDF in ROS or you can have your own XML or json file, you just need to care and not use any hard-coded value. [...] This gives you already some flexibility.”* 
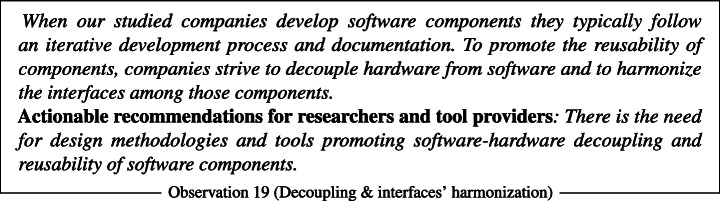


##### Inter-department Communication

An approach followed by both companies to promote the development of robotic skills and their reusability within the company is the dialogue between departments in charge of the development of robotic platforms. In the case of Blue Ocean, to assess whether developing a feature for a certain robot is feasible, a group of experts consisting of a brand manager, a project technical leader, a business owner from another project of the company, and a user-requirements specialist is assembled. Once the feasibility of the development is evaluated, a group of developers that follow SAFe-like^23^ processes is assigned to the task. To promote collaboration among the company’s projects, practitioners at Blue Ocean maintain oral-based communication, i.e., the architects of each of the robot projects meet regularly to keep each other synchronized and updated on development decisions. The company prioritizes oral communication over written communication since the latter gets quickly updated. Collaboration at the company follows a quick pace and practitioners keep informed using communication platforms such as Slack.

In the case of PAL Robotics, they hold several types of meetings. First, they hold weekly Scrum meetings to which “areas” (i.e., workshops & mechanics & electronics, software, business) of the company participate separately. They also have bi-weekly meetings to which the company’s business units attend separately. 
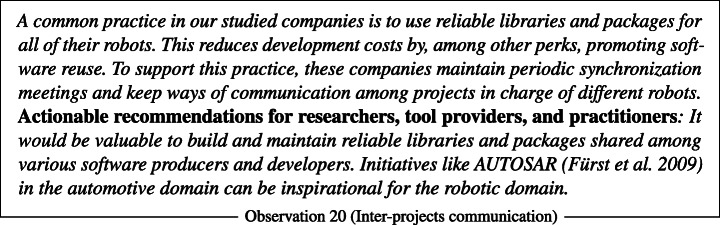


#### For Hardware Customization

In some cases, it is desirable to make hardware customizable, either because the company wants to provide such flexibility to customers or to ease possible future hardware updates.

The points of view of both companies on this topic are quite different, since, as explained in Section 3, the scope of both companies differs with respect to their products. Blue Ocean does not aim to support hardware customization since their products are professional service robots whose hardware is fixed once released to the market. According to two interviewees from Blue Ocean, this customization is technically possible, but they try to avoid it to reduce costs and effort I6: *“It is technically feasible. In UVD, we are using camera A. Let’s say, that then the product goes to the market and then we find out this camera A is not anymore in production. We can do camera B, and then we can do everything that is needed to deploy camera B, but it’s usually very expensive because it is not about just doing a piece of software, that is the easy part. We usually have to modify mechanical parts, covers, which implies new molds and there is a lot of costs associated with hardware changes.”* Instead, they try to stick to hardware components they know they work after studying and testing their performance. Special cases of customization happen in Blue Ocean at the product-level. Then, instead of creating variants of the same product, they create two different products—e.g., the company provides two types of GoBe robots.

On the other hand, PAL Robotics aims to provide products tailored to their customers by allowing the customization of their robots. This is a core policy within the company that gives rise to variability stemming from such customization. In this section, we discuss the following strategies identified from the interviews: (i) developing a **unified and customizable codebase**, (ii) to **harmonize interfaces**, and (iiii) to provide **add-ons**.

##### Unified and Customizable Codebase

PAL Robotics has invested time to develop a system that allows the easy integration of new hardware components by creating a unified codebase. All of their robots have the same core of code, which hugely simplifies code development and maintenance. However, they also make assumptions when developing this code. For instance, when they first developed TIAGo, they assumed it would only have one arm, but at the time of releasing TIAGo++^24^ (i.e., a new model of TIAGo with two arms), they had to revisit the code, which is considered a costly task by the company. To avoid such problems, the company now develops its code in a parameterizable way to also ease the reuse of its core code for future robot models. Besides parameterization, the success of PAL Robotics’ unified codebase relies on the harmonization of interfaces and on automatically generating configuration files that deal with interfaces, libraries, and dependencies.

The parametrization options are maintained in configuration files, in YAML format. I2: *“We try to put as much as possible in configuration files because they are very easy to see what has changed. They are centralizing one or a couple of locations, and you can make changes without recompiling your code, which is a pain. So yeah, I think they are quite cost effective. The parameter settings can be changed before and during runtime with a graphical user interface that allows the user to inspect and change the value of every parameter. In addition, there is also support for automated parameter-runing at runtime, especially for those parameters that are generally heavily affected by environment conditions (e.g., temporary drift compensation).”*

PAL Robotics interviewees consider that despite the time and effort it took them to reach the state of maturity of their hardware customization strategies and mechanisms it hugely simplifies managing hardware-customization-related variability. Therefore, they consider their current hardware-customization strategies and mechanisms cost-effective. I1: *“Most of the software is designed in such a way that it’s easy to extend [...] Quite often, inside our software, you find pieces that are not yet used but exist just in order to be able to integrate something new like [...] new planers for navigation [...] or the whole body control stack.”*

Despite the advances made by PAL Robotics to ease the processes related to hardware customization, there still exist limitations, mostly related to the time such customization would take. If a requirement from a customer is considered not feasible or not realistic from the company’s point of view, it is communicated to the customer. The process for deciding whether a requirement is feasible or not is not formalized and typically includes a discussion among developers I2: *“We do not have a formal process but typically it involves discussing internally with some fellow developers [...] It’s not a formal process, its common sense and corroboration from your partners.”* Another limitation is related to the physical connections of sensors and actuators. If, for instance, a specific camera model requested from a customer requires a voltage supply not provided by the panel of a robot, the camera cannot be integrated.

##### Harmonized Interfaces

To promote the reusability of skills among platforms as well as hardware customization and software development, a common practice applied by both companies is to harmonize interfaces of software components of their architectures—see the discussion earlier in this section, in the context of robotic skills development. This strategy is especially important at PAL Robotics, where hardware customization is one of the main goals. For instance, at PAL Robotics, robotic hand manipulators—either a five-finger hand or a gripper—use the same harmonized interface so they can be easily replaced without modifying the software control system of the robot. I5: *“At the end of the day, it’s about having clear interfaces and contracts. [...] Given these requirements you can build software that is reusable.”* 
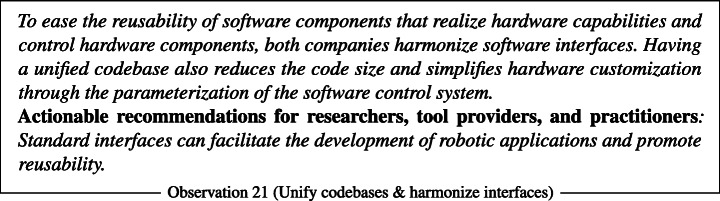


##### Add-ons

One Blue Ocean interviewee indicates that an alternative they use to hardware customization is hardware add-ons. For example, a common addition to their products is dock stations.

### Hardware: Mechanisms

Mechanisms applied by both companies for managing hardware variability are mainly focused on control system design and developing and maintaining inter-usable skills among robotic platforms.

#### For Control System Design

This section discusses the mechanisms used by the studied companies to design the system in charge of executing the robots’ behaviors. Concretely, we discuss (i) **software architectures**, (ii) **middleware**, (iii) **ROS_control**, (iv) **standards and safety layers**, and (v) **version control**.

##### Software Architectures

Robot control systems are often developed and structured adhering to a software architecture (García et al. 2018; Kortenkamp et al. 2016; Ahmad and Babar 2016). PAL Robotics’s robots follow a unique reference architecture, which is realized as a platform that contains the unified codebase of their robots (Obs. 21). The platform consists of modular and reusable software components and their interfaces. At Blue Ocean, each product is developed adhering to a unique architecture, most commonly being layered and component-based. A company policy is to reuse as much software (Obs. 26) and mechanisms as possible, which may somewhat constraint the architectures.

##### Middleware

Robotics companies usually rely on frameworks and middleware to support the building of their software systems, as found by García et al. (2020). According to most of our interviewees, robotics software development has been hugely simplified since ROS, which promotes software engineering best practices like modularity and reusability. ROS provides an infrastructure, drivers for most sensors and actuators, and hardware abstractions. ROS helps managing variability stemming from the hardware (providing hardware abstractions and drivers) and the robotic skills, which are modularized as reusable software components. Part of the benefits of ROS is not related to the middleware itself but to other factors like its community and existing ecosystem. I6: *“To me, ROS middleware is like 10% and 90% of ROS are other things. Like [...] the implementation that it has of a service-oriented architecture, creating nodes as packages, all the building infrastructure, and the community.”* A possible outcome of the usage of ROS at both companies is that they both use Linux-based operating systems. Ubuntu and ROS distributions^25^ are drivers of variability that we studied in the first stage of our study (García et al. 2019b). It is worth remarking that PAL Robotics also uses OROCOS (Bruyninckx 2001) for components with real-time requirements.

##### ROS_control

26 It is a mechanism for ROS users that helps software development by abstracting hardware details and providing standard interfaces for the drivers of actuators and sensors, which make controllers robot-agnostic. This simplifies the variability management of hardware from the robot control system point of view. I4: *“From this, we can abstract the type of model that we are using, the type of communication bus, the number of models, the kinematics of the robots because this is embedded in the robot description file.”* 
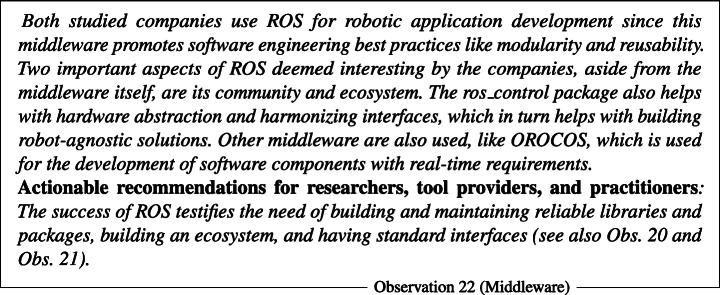


##### Standards and Safety Layers

Another important characteristic of robot control systems is that robots must be designed according to some certifications and standards. For instance, safety standards have an impact on the embodiment of the robot, since the mechanical part, sensors, and hardware must adhere to specific certifications. The standards also concern the operating environment; for instance, if the robot must interact with humans special certifications apply. Companies need then to manage variability stemming from hardware components or specific robotic skills affected by safety standards. To manage such variability, both studied companies integrate safety layers that override some autonomy aspects of the robot to conform to the standards and other safety measures I9: *“We do have a safety certified layer that overrides all the autonomy of the robot in case we detect any danger. Imagine a Roomba or some of these cleaning robots[...] You have those drop-off sensors so that the Roomba does not fall from the stairs. They just override the control algorithm that’s running the autonomy cleaning procedure.”* 
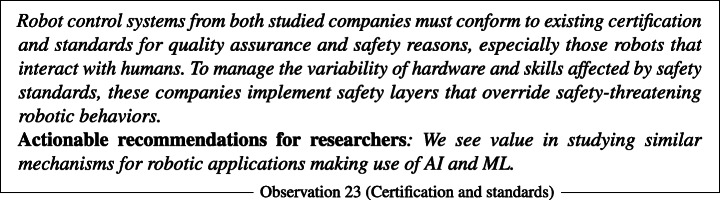


##### Version Control

Both companies make use of version control mechanisms for code development and maintenance of their robot control systems. They both rely on Git-based mechanisms.

PAL Robotics follows a clone&own strategy, using a branching policy similar to ROS’s—i.e., developers create a new branch for each new ROS version. PAL Robotics developers use a single branch “master” to simplify maintenance. However, when it is not possible to keep backward maintainability—if, for instance, interfaces or data of earlier versions cannot be successfully used by newer versions—the new code must be adapted and developers in PAL Robotics create a new development branch. An example of such adaptation is some new code to adapt from ROS Indigo to ROS Kinetic. Then, the branch major version is changed (e.g., from 1.2.3 to 2.0.0). Finally, developers from PAL Robotics leave the old default branch with the name of the old development branch (e.g., “indigo-devel” branch), leaving it in the state of backward compatible with the newer branch. Branches are maintained forever and backward-compatible bug fixes are conducted for all the branches if possible and necessary.

Blue Ocean uses Bitbucket and the Atlassian universe and work with a unique combination of ROS and Ubuntu distributions, that is, ROS Melodic with Ubuntu 18.04. Blue Ocean maintains a forked repository of rosdistro,^27^ which contains the packages and dependencies used for specific ROS distributions. The company customizes the rosdistro repository. For example, by substituting the URLs of the repositories for the software packages of the original ROS distribution with the URLs of company-developed repositories. To modify a repository, the company creates a branch and a pull request, which needs to be approved by one or two developers. The company uses an in-house tool that allows choosing specific commits/tags of each of their software packages from a superbundle generated by the same tool. The tool extracts the system dependencies and generates a deb file that contains their customized version of ROS. Blue Ocean works with a private advanced package tool (APT) to which developers publish the binaries and to which robots have access. Blue Ocean’s robots then access the APT to retrieve the software packages required for their operation. 
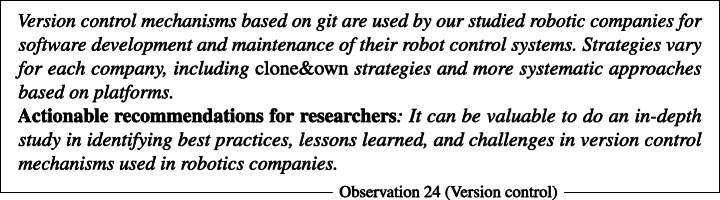


#### For Inter-usable Skills Among Platforms

Both companies strive to make skills and components as reusable as possible. The goal is to ease development efforts by making skills usable by as many robots as possible within the company. This includes robots that are developed by different projects I3: *“From the beginning, we choose to have the same software layers in all the robots. So pretty much, once the component is working in one of the platforms, you can make it work in another platform right away.”* An important case of skill reuse among projects is navigation. Companies strive to abstract the locomotion details of each robot: There are substantial differences between a bipedal or a wheeled robot. However, there are limitations to the reusability of software components and skills, being the main reasons:

- The layers of the architecture which are closer to hardware may differ among robots—i.e., robots providing different services or operating in different environments often equip different actuators.
- The robot-specific missions vary among robots that provide different services. To accomplish such missions, robots usually perform specialized skills—e.g., the PTR robot lifts patients and moves them to different locations, which is completely different from what a GoBe robot is intended to accomplish.

To deal with these limitations and manage variability, both companies make use of different mechanisms. Namely, we discuss (i) **configuration files and parameters** (discussed previously, in Section 5.2), (ii) **in-house tools**, and (iii) **libraries**.

##### In-house Tools

At PAL Robotics, practitioners use an in-house tool that automatically generates configuration files and deploys them within the robot during its installation (García et al. 2019b). 
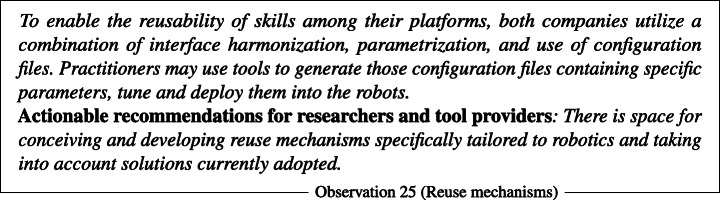


##### Libraries

Software modularization is pursued by both companies to promote their software reusability. It is accomplished by the studied companies by following a component-based software development approach, which is enforced by the usage of ROS. In line with the implementation of ROS is the usage of libraries and packages, which in turn promote modularity and reusability in the robotic applications of both companies. These libraries are either publicly available (e.g., BehaviorTree.CPP^28^ and py_trees^29^) or developed in-house. One policy from Blue Ocean is to make the implementation of packages and libraries common to all projects within the company. For instance, libraries and packages concerning the cognitive layer (i.e., py_trees as the mission specification mechanism) and the planners are common among projects. This policy simplifies decision-taking, the harmonization of interfaces, and variability management within the company but at the same time may constrain the design of the robot control systems. 
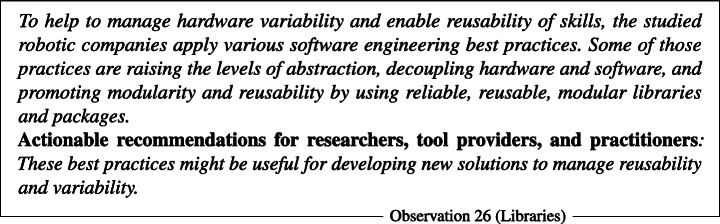


### Mission: Strategies

Our studied companies apply one strategy to manage variability in missions, namely **generic missions**.

#### Generic Missions

Similar to the generic configurations discussed in Section 5.1 (Obs. 11), interviewees from both companies detailed their strategy to conceive generic missions that can be applied to a wide range of scenarios without requiring major modifications. Normally, scenarios where professional service robots operate are quite specific; for instance, a UVD robot’s most common type of environment is hospitals and therefore many features of the environment are standardized and known beforehand—e.g., the maximum slope in the corridors. An example of a generic mission is the following. A UVD robot that operates in a hospital idly waits in its charging dock until it is commanded to disinfect a room. This mission encodes a set of tasks or sub-goals, including navigating from the charging dock to the target room, going through the room’s door, and interacting with an operator to ensure that there are no unexpected obstacles that would impede the robot’s operation and that no human is inside the room while disinfecting. These generic missions use specific parameter values (e.g., the robot’s speed while navigating the hospital corridors or while going through a door) that are used at run-time by the robot. The goal of these generic missions is to simplify the mission specification process and avoid parameter fine-tuning during this process. However, their conception and lifting their success rate is considered a challenge by the company, as we will explain in Section 6. 
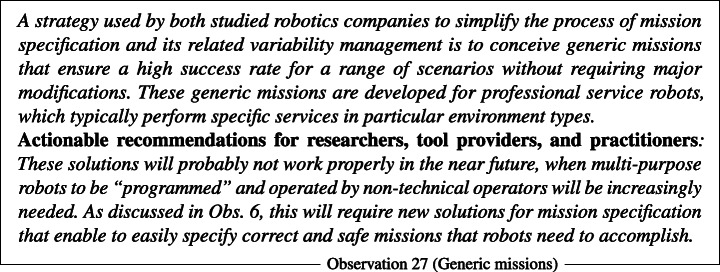


### Mission: Mechanisms

In the following, we detail mechanisms utilized by the studied companies to manage variability that stems from robotic missions, concretely: (i) **state machines**, (ii) **behavior trees**, (iii) **flowcharts**, (iv) **task** **frameworks**, (v) **GUIs**, and (vi) **navigation frameworks**.

**Finite-state machines (FSMs)** are the main mechanism that PAL Robotics uses and provides to their customers to specify missions. Concretely, they make use of an in-house implementation of SMACH, a Python-based library from ROS’s ecosystem used to specify robot behaviors using hierarchical state machines. SMACH is also used to define the adaptation rules for their robotic applications (Obs. 15).

Different interviewees come to a different assessment of state machines as a means of implementing variability in missions. Several PAL Robotics interviewees consider them user-friendly. While they acknowledge that FSMs are not suitable for non-technical operators due to their complexity, they assume their customers to be technically skilled. PAL Robotics’s CTO considers that FSMs are hard to comprehend due to their associated learning curve. Furthermore, they express concern about the scalability of FSMs, which quickly become very complex when adding conditional and nested states, leading to increased maintenance costs. A similar concern is shared by our interviewees from Blue Ocean, describing situations where a large amount of dynamic variability needs to be supported. In line with this sentiment, one engineer at PAL Robotics mentions that state machines are quite often used for demos and small projects.

**Behavior trees** are the underlying mechanism used by Blue Ocean to define missions for their robots. The company uses either py_trees or its derivative BehaviorTree.CPP, both domain-specific languages realized as libraries based in Python and C++, respectively. Blue Ocean uses an in-house framework to enforce the trees’ topology and a template for the action providers—e.g., *GoToPosition*. The behavior trees are built using this framework and are generic for all clients. To customize their missions, Blue Ocean creates a database for each customer that contains customer-specific information. When loaded into the cognition layer of the robots, the behavior tree can query the customer’s database and configure itself based on that information.

Compared to FSMs, behavior trees are on a higher abstraction level, focusing on high-level actions that are implemented in the code using an asynchronous request-reply pattern. Interviewees at both considered companies point out greater flexibility and reuse potential when using behavior trees for supporting variability. One interviewee illustrates this in an elaborate example:

I6: *“What we are using the most is that we can prune and inject trees in the run time. For example, in the predefined disinfection mode, let’s say we have five disinfection positions where the robot has to go. Now, let’s say that the robot goes to the first one and fails the second and third ones: suddenly someone tries to enter the room, and the disinfection is interrupted. Now, the operator has to decide what to do. You can repeat all the disinfection positions, repeat the failing positions or continue the disinfection as it was before. We don’t know what is the right answer from the robot’s perspective. The operator might not want to go back to the other two points, we might know the distance or we don’t know the urgency of that operator. Maybe a person is coming, and they need to be in the room right now. We give the person the option of selecting what to do next. Based on the selection of the user, then we inject the tree accordingly. We can reconfigure, prune, and inject trees and sub-trees. That’s why we prefer to use behavior trees rather than state machines, because state machines are much stricter.”*

#### Flowcharts

One of the first steps to design a product at Blue Ocean is specifying the scenarios (Obs. 10) that describe the intended operation of the product using flowcharts, internally known as “technical workflows.” These workflows also detail the required functionalities for the different software levels of their robots. This encompasses from mission specification to the actual code implementation in low-level layers of software and its expected behavior in certain contexts. Therefore, this mechanism is cross-cutting across all the drivers of variability studied in this paper, and orthogonal to the more technical mission specification mechanism being used, such as FSMs or behavior trees. The technical workflows are used by the company to infer the requirements of a product, which need then to be evaluated to assess their business value. Concretely, the mission specification aspects of the workflows are defined in collaboration with the customer, from which the requirements are inferred. Once the mission workflow of a robot is properly defined, it is translated into behavior trees.

#### Task Frameworks

PAL Robotics also uses and provides to their customers a task framework, which is used to manage the scheduling of simple, highly repetitive tasks (e.g., going from point A to point B). They also provide a graphical user interface for the operator to schedule these tasks, including timing and generating maps.

A future goal for PAL Robotics is to develop a task planner where high-level missions can be easily defined and which automatically generates a specification based on a target mechanism, namely behavior trees or state machines. In this way, the company would avoid hard-coding the missions, which would enhance its mission-related variability management while simplifying mission specification for its customers.

#### GUIs

Besides the infrastructure provided by the company, the complexity of behavior trees is still high for non-technical operators, and therefore this specification is hidden from them. Instead, the operator will be presented with a graphical interface or GUI installed on a tablet. The GUI presents rather simple information to the operator, who just needs to push a button to start the mission. The operator may also set a few parameters—e.g., in the case of a UVD robot, the room to disinfect—to configure the mission to their needs. Note that non-technical operators are not able to change the underlying mission-specification mechanism (i.e., a behavior tree) but rather to *configure* it. 
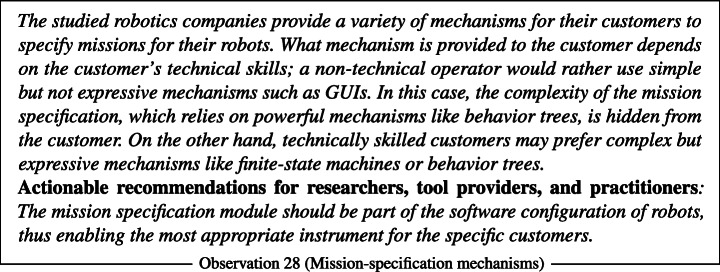


#### Navigation Frameworks

Regarding the navigation aspect of mission specification—inherent to mobile robots—, both companies use frameworks that are made common to all their robots. Blue Ocean works with an in-house solution that implements their custom *contextual navigation* mechanism (explained in Section 5.2.2). PAL Robotics relies on move_base,^30^ a well-known package for navigation-related tasks from ROS’s ecosystem. move_base performs a navigation task by combining two planners: a global and a local one. The global planner solves a global path-finding problem, whereas the local planner provides adjustments based on dynamic sensor input (e.g., collision avoidance). Global and local planners can be exchanged freely, as long as they implement the interfaces provided by move_base.

### Variability Management Practices in the Literature

The studied papers aim at promoting the adoption of effective software engineering methods for developing robotics software. Papers from Table 6 discuss engineering paradigms such as Model-Driven Engineering (MDE) (Brugali and Gherardi 2016; Goldsby and Cheng 2008; Silva et al. 2013), Software Product Line Engineering (Brugali and Hochgeschwender 2018; Lotz et al. 2013), Software Frameworks (Kimour et al. 2009), and Component-Based Software Engineering (Niemczyk and Geihs 2015).

**Table 6:** Variability management in the literature (RQ2)

			E^a^	H^b^	M^c^
Ground robots	P1	Lee et al. (2006)			∙
	P2	Álvarez et al. (2006)	∙	∙	∙
	P3	Kimour et al. (2009)	∙	∙	∙
	P4	Steck and Schlegel (2011)	∙	∙	∙
	P5	Lotz et al. (2013)	∙	∙	∙
	P6	Brugali and Gherardi (2016)	∙	∙	∙
	P7	Brugali and Valota (2016)	∙	∙	∙
	P8	Brugali and Hochgeschwender (2017)	∙	∙	∙
	P9	Brugali and Hochgeschwender (2018)	∙	∙	∙
	P10	Brugali et al. (2018)	∙	∙	∙
	P11	Rollenhagen et al. (2019)	∙	∙	∙
	P12	Wirkus et al. (2020)			∙
	P13	Seiger et al. (2015)			∙
	P14	Niemczyk and Geihs (2015)	∙	∙	∙
	P15	Goldsby and Cheng (2008)			∙
	P16	Saglietti and Meitner (2016)			∙
	P17	Buchmann et al. (2015)		∙	
UAVs	P18	Brown et al. (2007)	∙	∙	∙
	P19	Steiner et al. (2013)	∙	∙	
	P20	Silva et al. (2013)	∙	∙	∙
	P21	Fragal et al. (2013)	∙	∙	∙
	P22	Ozdemir et al. (2014)	∙	∙	∙
	P24	Czerniejewski et al. (2016)	∙	∙	∙
	P25	Feng et al. (2015)			∙
	P26	Braga et al. (2012)	∙	∙	∙
	P27	Olaechea et al. (2018)	∙	∙	∙
	P28	Brooks and Iagnemma (2009)	∙		
	P29	Pant et al. (2015)	∙	∙	∙

^a^ Environment variability

^b^ Hardware variability

^c^ Mission variability

A few papers address the interplay of the various drivers of variability in robotics and propose approaches for their effective management. Brugali and Valota (2016) present an MDE approach that allows engineers to model both variability in robot functionalities (packaged in reusable software components) and in application requirements (related to hardware, environment, and task variability). The HyperFlex tool supports the automatic configuration of system functionality from a selection of application requirements. Steck and Schlegel (2011) developed an MDE approach for runtime automatic selection of different execution variants of a robot control system. The proposed approach exploits several variability models related to component-based architectures and task plans (i.e., missions).

A limited number of papers do not specifically address software variability in robotics, but present general-purpose variability management approaches that are exemplified using a case study in robotics, as reported by Lee et al. (2006) and Olaechea et al. (2018). 
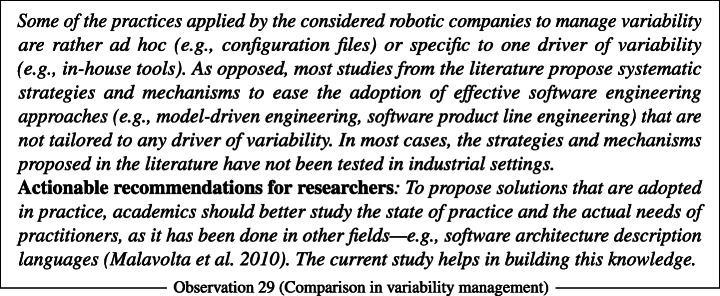


## Variability-Related Challenges (RQ3)

We now describe the challenges we identified that practitioners from both studied companies face, structured into subsections for each driver of variability. We then present the related findings from our SLR and an observation that details the triangulation of these findings with the results from the interviews.

### Environment

Our interviewees discussed a variety of challenges related to their robots’ operating environments, from which we identified (i) **conceiving generic solutions for various scenarios**, (ii) **developing parametric configurations**, and (iii) **the installation process**.

#### Conceiving Generic Solutions for Various Scenarios

Both companies collect feedback from their customers to identify the causes of failures for their robotic applications. For instance, PAL Robotics records data from scenarios where their customers reported failures, which triggers an update in the robots’ software to fix derived issues and make the robots more robust. However, PAL Robotics practitioners realized that it is intractable in the long term to manage several configuration files (typically yaml and launchfiles, see Section 5.2) for each scenario. I2: *“These configurations [...] are cost-effective but they are not cheap either. So you cannot configure everything because you would have endless parameters for everything and then you end up with something that no one knows how to use.”* Similarly, a Blue Ocean interviewee claims that trying to cover every possible corner case of robot operation using adaptation is unfeasible. Therefore, a goal for both companies is to find the “sweet spot” where robots succeed their missions without handling every potential event. This strategy to manage environment variability (either through configuration files or adaptation rules) requires of generic solutions that fit most scenarios and allow robotic applications to accomplish their missions with a high success rate. However, according to interviewees from both companies, conceiving such solutions and finding a good balance between performance and error-handling complexity is challenging.

To conceive generic solutions, companies need to first carefully study the environments where their robots may operate, which requires several iterations with the customer. Then, companies follow different strategies. At Blue Ocean, developers first describe missions and scenarios using workflows in collaboration with customers (Obs. 28), which they use to specify the context and events their robots are expected to adapt to. A second step, common for both companies, is performing several testing iterations to validate the configuration of the robotic application and its robustness. 
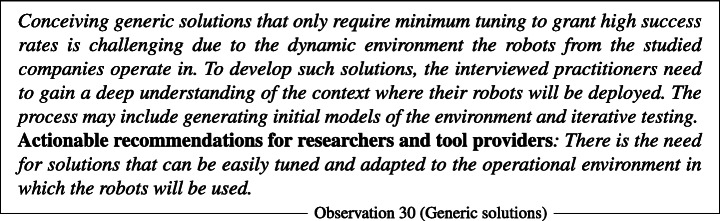


#### Developing Parametric Configurations

Some scenarios make generic configurations unfeasible, either due to the scenario’s complexity or uniqueness. For these scenarios, companies create sets of configuration files that use parameters (Obs. 12). Companies elicit and model the parameters needed to adapt their generic applications from the customers. Also, some environmental features are unique for certain scenarios. For instance, practitioners from Blue Ocean explain that hospital rooms are different around the world and that sizes and shapes of toilets change for every country. Our studied companies choose to hard-code those special environmental features into the system, increasing the complexity of variability management. 
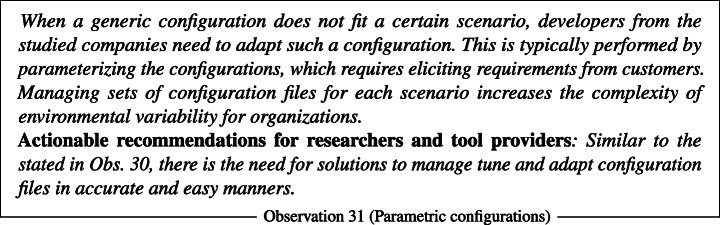


#### Performing the Installation Process

Even when companies are able to provide generic solutions that fit the customer requirements, the installation process explained in Section 5.1 (Obs. 9) needs to be performed. This consumes time and resources from organizations. Blue Ocean interviewees mention that they are working on APIs that will guide customers through this process, allowing them to install the robots in their environments by themselves. From PAL Robotics, two practitioners mention that their goal and the main challenge in this regard is to ship robots that are able to adapt to a known environment. This requires eliciting environmental features from the customer before shipping the robot but also dealing with dynamic variability. This remains an open challenge for PAL Robotics, as documented in our previous study (García et al. 2019b).

According to one interviewee from Blue Ocean, the lack of standardized solutions (e.g., tools and interfaces) impedes that an operator such as a system integrator could perform the installation process.

### Hardware

According to our interviewees, there exist many challenges related to hardware variability in robotics, namely (i) **conceiving generic solutions for different robots**, (ii) **hardware-driven assumptions**, (iii) **achieving reliability of variant-rich systems**, (iv) **multiple options for hardware design**, (v) **lack of standardized hardware options**, (vi) **performing integration**, (vii) **aligning conventions for hardware**, (viii) **trade-offs**, (ix) **handling real-time components**, and (x) **influence from environment.**

#### Conceiving Generic Solutions for Different Robots

In Sec. 5.1 and 6.1 (Obs. 11, 30), as well as in Section 5.5 (Obs. 27), we discussed how companies strive to create generic and reusable configurations that have a high success rate while avoiding continuous configuration. On top of that, to make those configurations of missions and scenarios truly generic, the studied companies invest resources in making such configurations robot-agnostic. To achieve that, companies need to raise the level of abstraction of software components that realize robotic functionalities (i.e., skills) to make them usable by different robots within the same company (Obs. 19, 25, 26). This simplifies variability management, but, according to one interviewee from PAL Robotics, complicates software development, as explained below.

#### Hardware-Driven Assumptions

Both studied companies strive to reuse their code for their robots’ control systems and skills as much as possible to simplify development and reduce time to market (Obs. 17, 18, 19, 20). However, assumptions made in the software based on the hardware design of the robots threaten its reusing. I2: *“If making a single robot to navigate in a crowded environment is already a tremendous task, making the same code work for any kind of robot [..] doesn’t make it simpler. It also limits you by not being able to make certain assumptions, which makes the code more verbose: you have to add more code to handle different scenarios.”* An example given by one interviewee from PAL Robotics is that they developed skills for TIAGo assuming that the robot has one arm. However, PAL Robotics has recently released a new model of the robot that has two arms (TIAGo++^24^), which required developers to refactor the code of their robots’ control systems to accommodate the new hardware design. This was complicated since the hardware-driven assumptions were not documented when the code was developed. The lack of documentation also hinders the maintainability of such code and the integration of other robotic platforms that may not share the same assumptions, according to this interviewee.

PAL Robotics’s current strategy to palliate this challenge is to parametrize their robot control system and skills to make them flexible to different platforms, as explained in Section 5.3 (Obs. 21, 25). 
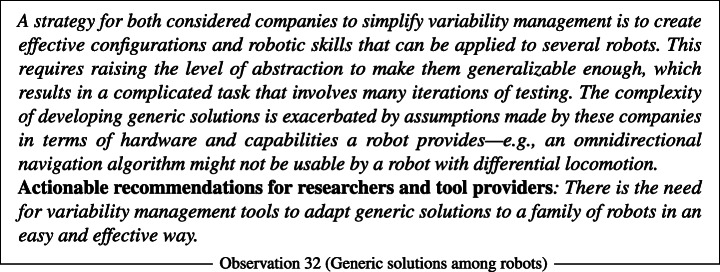


#### Achieving Reliability of Variant-Rich Systems

Building reliable variant-rich systems is one of PAL Robotics’s most pressing challenges, according to one of their software engineers. Some of the company’s robots have more than 40 variants. To ensure a robust operation of such variant-rich products, PAL Robotics tests independently all the low-level features of their system—e.g., the different end-effectors a TIAGo may equip, see Figs. 1 and 4. In this way, developers can test the performance of missions—higher in the abstraction layers—without conducting separate tests for each variant. I2: *“Once you go higher in the abstraction layers you have to assume that the lower layers are behaving properly, so when you are doing a grasping experiment you don’t need to repeat the same experiment with three different cameras, four lasers, and one dozen combinations for the arm.”* 
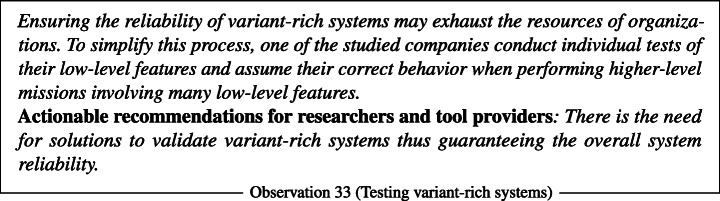


#### Multiple Options for Hardware Design

One interviewee from each company consider the robot hardware design the most challenging issue to deal with regarding robotics variability. According to these interviewees, there is a vast supply of electrical components, sensors, and actuators that could be integrated into the robot hardware design, which directly impacts the robot control system and its variability. Managing this variability becomes challenging when the selection of components, sensors, and actuators is often decided upon developers’ personal preferences and backgrounds. This is exacerbated by the lack of standard options, which also affects the reliability of the product.

#### Lack of Standardized Hardware Options

One interviewee from each company argue that having standards would immensely simplify the process of integration at many levels of robotics, ranging from sensors, via their drivers and necessary software for the robot control system, to software components that make use of them. I1: *“What a camera does in one way, another camera does in a completely different way [...] and you have to rebuild half your software to take into account the possibility of having one camera or the other.”*

#### Integration

A challenge highlighted by one interviewee from Blue Ocean is the complexity of the integration of hardware and software components tailored to robotic capabilities due to the existing variety of electronics, mechanics, protocols, and software. The vast supply of hardware and personal preferences complicates the integration for each development project within the company if not standardized or harmonized. Similarly, the integration of software in charge of controlling the variety of electronics, functionalities, mechanics, and protocols is also challenging. A robotic software application is typically developed following a component-based approach (Obs. 22) (Brugali and Scandurra 2009; Ahmad and Babar 2016) where each software component may be developed by a different person—e.g., a control engineer may have developed the motion control aspects of the robot while a software engineer carries on with the cognitive layer. These components need to be integrated into a common platform. This, according to our interviewees, consumes a high amount of effort. Interviewees from both companies explain that they rely on ROS for software integration since it simplifies this task by providing standardized interfaces and message types (Obs. 22). 
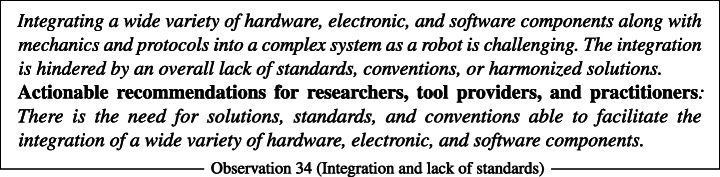


#### Trade-offs

We noticed a series of trade-offs related to hardware variability that are perceived as challenges by our interviewees, which we synthesize in the following.

*Costs versus time*.: Some hardware elections influence the integration of the whole robot in terms of time, according to one interviewee from Blue Ocean. For instance, a more expensive sensor could have an existing ROS driver, which would make the sensor integration and software development much faster.
*Flexibility and costs versus performance and usability*.: Making a robotic application specialized to certain scenarios could reduce its complexity or improve its success rate, as discussed by one interviewee from each company. However, as discussed in Obs. 11, 27, 30, and 32, generic solutions help simplifying variability management, reducing the company’s costs related to developing, maintaining, and tuning of configuration options. For instance, to make navigation skills as reusable as possible, the locomotion mechanisms are abstracted, and generic or harmonized interfaces are provided. Still, implementation difficulties may arise if the same interfaces are provided for ground, aerial or underwater robots, which operate using different parameters—e.g., a ground robot does not necessarily need to care about the aircraft principal axes of yaw, pitch, and roll. Companies try to find a balance between generalization and performance from tailored solutions.

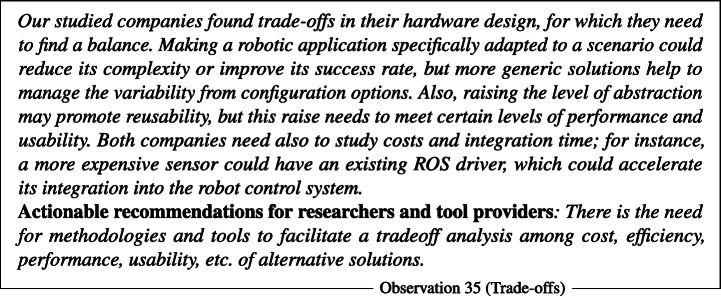


#### Handling Real-Time Components

Achieving real-time performance of the robot control system is crucial for robots that operate or collaborate with humans (Rouxel et al. 2020), as is the case of most service robots from our studied companies. Since ROS does not handle real-time, developers at PAL Robotics use OROCOS to develop and manage real-time constrained components. Besides the complexity of controlling real-time systems, PAL Robotics needs also to manage the variability stemming from the usage of two middleware (i.e., ROS and OROCOS) to develop and control their robot control systems.

#### Influence from the Environment

The hardware a robot equips is strongly influenced by the environment where it is intended to operate (Obs. 2). As an example, a robot operating outdoors may incorporate GNSS sensors to improve its self-localization capabilities, but the same sensors will not work indoors. Moreover, there exist LIDAR sensors suitable only for indoor scenarios and some that also work outdoors. Similarly, a robot navigating on rough surfaces may experience problems with sensors using a USB connection, more suitable for indoor environments, due to vibration. Adapting to such requirements increases hardware variability and its management complexity.

### Mission

Challenges our interviewees normally face concerning robotic missions are (i) **mission specification**, and (ii) **promoting user friendliness**.

#### Mission Specification

Missions for service robots typically fulfill requirements requested by the customer, as explained in Sections 4.3 and 5.6. Associating high-level user requirements from customers to individual robot configurations is deemed as a challenge by two interviewees from Blue Ocean.

One interviewee from PAL Robotics and two from Blue Ocean rate generating the mission that describes the set of goals a robot must achieve while being able to deal with the environment as the most important challenge for robotics variability management. Among the main issues is the uncertainty present in the environments where service robots operate (Obs. 3). That is, dealing with unexpected events or failure of systems complicates the specification of missions and managing their variability.

One interviewee from Blue Ocean highlights the impact of time-sensitive constraints, which also need to be managed during mission specification. For instance, timing may vary in a social context where robots need to move carefully to not harm humans or in an industrial scenario, where timing is intrinsically related to performance. Related to the context, missions that fulfill the specification from customers must meet existing standards of safety.

PAL Robotics’s CTO states that a high-level tool that explores the existing skills set of a system and automatically builds execution graphs that in turn can self-reconfigure at runtime based on requirements and the environment would be highly beneficial for their organization. As an example of reconfiguration, a robot could lock some arm joints to reduce power consumption in case that no grasping task is required. Similarly, if a robot’s motor stops working properly, it may deliberate whether is more efficient to keep working in a slower way to finish the mission or to call for a failure. 
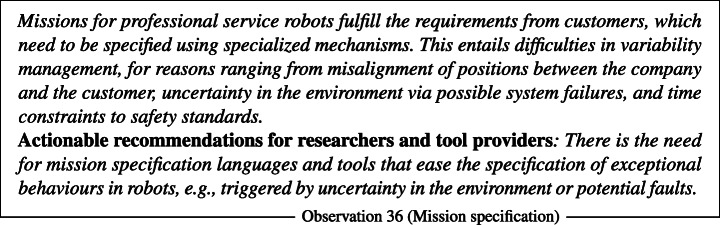


#### Promoting User-Friendliness

Two interviewees from each company state that promoting user-friendliness in their mission specification tools is challenging. The common goal for both companies of developing an expressive tool that supports the operator by providing simple and enough information during mission execution entails many difficulties related to variability management. For non-technical operators, the tool should be able to provide an easy-to-understand interface and automatically deal with the variability from the underlying mission specification mechanisms (i.e., behavior trees in Blue Ocean and finite-state machines in PAL Robotics). That is, the tool should configure such underlying mechanisms based on the mission specification and the setting of parameters specified by the customer. In summary, the tool should support and keep the user informed while hiding the unnecessarily complex back end of the tooling. To do so, the studied xcompanies rely on the specification of generic missions that allow reliable mission execution in certain scenarios with minimum configuration (Obs. 27).

Moreover, the companies consider that users should not be constrained by dealing with operating system distributions, versions of ROS, or the compiler and libraries. According to PAL Robotics’s CTO, developing a graphical tool able to generate code without the mentioned constraints is a future business goal for the company.

Finally, an interviewee from Blue Ocean states that they are working on user-friendly tools that will allow operators to perform mission-related tasks like mapping the environment and setting regions of interest (Obs. 9). This would reduce the complexity of variability management and the installation process for companies. In the context of the two studied companies, user-friendliness alludes to the effort they carry out to make as accessible and simple as possible their complex products and processes, especially for non-expert users. 
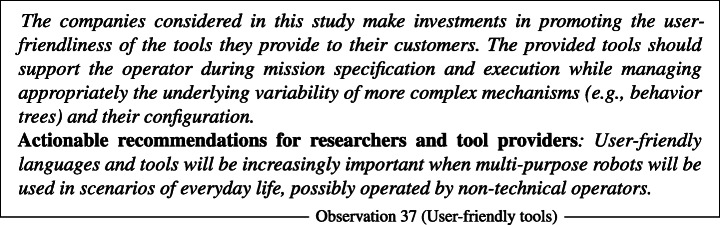


### Variability-Related Challenges in the Literature

Only a few papers (11 out of 30) from our SLR discuss challenges related to the adoption of variability management approaches in real-world scenarios, as listed in Table 7. The most significant challenges are related to the difficulties of (i) recreating in simulation the complexity found in variable real-world deployments, as proposed by Czerniejewski et al. (2016); (ii) managing the combinatorial explosion of product configurations in UAV product line development, as proposed by Olaechea et al. (2018), and of testing and certifying them, as might be found in Braga et al. (2012), Steiner et al. (2013); and (iii) associating high-level user requirements to individual robot configurations, as proposed by Duncan and Murphy (2017). In addition, Pant et al. (2015) discuss how variability related to the type of sensor induces a variability in the computational cost and accuracy of robot functionality. 
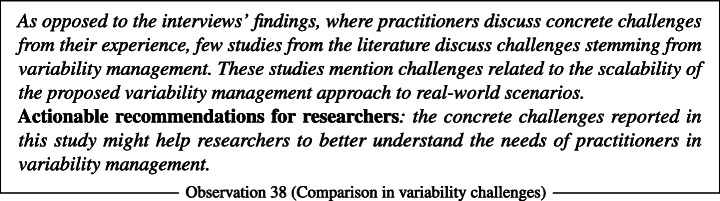

**Table 7:** Variability challenges in the literature (RQ3)

			E^a^	H^b^	M^c^
Ground robots	P6	Brugali and Gherardi (2016)	∙	∙	∙
	P10	Brugali et al. (2018)	∙	∙	∙
	P13	Seiger et al. (2015)			∙
UAVs	P19	Steiner et al. (2013)	∙	∙	
	P22	Ozdemir et al. (2014)	∙	∙	∙
	P24	Czerniejewski et al. (2016)	∙	∙	∙
	P26	Braga et al. (2012)	∙	∙	∙
	P27	Olaechea et al. (2018)	∙	∙	∙
	P28	Brooks and Iagnemma (2009)	∙		
	P29	Pant et al. (2015)		∙	
	P30	Duncan and Murphy (2017)			∙

^a^ Environment variability

^b^ Hardware variability

^c^ Mission variability

## Discussion

We now discuss and compare our findings among the interviews and the SLR, specifically: (i) drivers of variability (Obs. 8), (ii) variability management practices (Obs. 29), and (iii) variability management challenges (Obs. 38). We also discuss our finding’s impact and the possible exploitation of the results of our investigation by summarizing them, proposing hypotheses for our observed phenomena, and giving recommendations to practitioners and researchers.

### Drivers of Variability

#### Observations

Almost every paper we studied in our SLR addresses one or more of the drivers of variability we identified. The characteristics of environment variability mostly discussed in the literature refer to the features and changing conditions of the scenarios where robots operate, similar to some of the findings from our interviews (Obs. 2). Some papers also mention managing the variability entailed to perform the same mission in several environments. According to our interviewees, this is performed by the studied companies using generic and parametrizable mission specifications (Obs. 27).

Only two papers of the SLR show evidence of dealing with environments populated by humans. They deal with human-robot collaboration in disaster scenarios (Niemczyk and Geihs 2015) and factory automation (Rollenhagen et al. 2019). As opposed, both of our studied companies need to manage the variability stemming from the inclusion of humans to the operating environments of their robots (Obs. 3). As opposed to our interviewees (Obs. 5), the literature does not focus on variability stemming from hardware-related customer requirements. Mission variability in the literature is mostly focused on the variety of missions and scenarios robots may perform within a concrete domain or set of scenarios, such as logistics, home entertainment or cleaning of ship-hull surfaces. Human-robot interaction is mentioned only in one study from the SLR, while this topic has been thoroughly discussed in the interviews (Obs. 7).

#### Hypotheses

We hypothesize that service robots are highly change-centric software systems for two main reasons. First, the variability of robotic software artifacts is driven by the evolution of the underlying technologies in mechanics, electronics, computer science, and cognitive sciences (Obs. 4 and 5). Second, service robotics is a research field that pursues ambitious goals, such as to “expect robots to function on their own with people and each other under whichever environmental conditions they happen to find themselves” (Sukhatme and Matarik 2002). This means that the variability of robotic software applications is driven by the complexity of everyday environments (Obs. 1 and 3) and by the potential uses of service robots for everyday tasks (Obs. 6). We believe that these two reasons reflect the different interests of industrial development and academic research and explain the different emphasis on the various variability drivers that emerged from the interviews and from the SLR.

#### Recommendations

Our analysis revealed that robotic variability is commonly expressed in fuzzy and ambiguous or project-specific terms, and this makes it hard to understand what functionality the robotic system being built must express (Obs. 8). We recommend researchers work on creating a common ontology to describe the variability drivers existing in service robotics following the steps of previous research groups as Olszewska et al. (2017) and Köster et al. (2016). This would require collaboration with practitioners who should provide personal experiences on the topic. This would result in the definition of a common language for expressing the variability in robotic technologies and in robotic requirements that can support robotics engineers in the development of new service robotic applications. Using a common ontology and language would promote the reuse of available technologies for solving common and recurrent design problems and favor the comparison and evaluation of robotic systems. With our current study, we made the first steps toward such an ontology and the enlargement of the current body-of-knowledge of variability in service robotics.

### Variability Management Practices

#### Observations

Most studies from the literature propose adopting effective software engineering practices to manage the variability of specific drivers. Examples are Model-Driven Engineering (MDE), Software Product Line Engineering (SPLE), Software Frameworks, and Component-Based Software Engineering (CBSE) (Obs. 29). Findings from our interviews show that companies also adhere to such software engineering practices, e.g., promoting reusability and modularity (Obs. 19, 21, 22, 26). On the other hand, we learned that our studied companies also developed strategies and mechanisms tailored to specific drivers of variability, like in-house tools (e.g., Obs. 25) and configuration files (Obs. 12).

#### Hypotheses

Systematic approaches and paradigms such as MDE and SPLE, which have been successfully implemented in other domains (e.g., automotive, avionics) are not currently used by robotic companies. Our insights give rise to the hypothesis that one of the chief reasons is the lack of awareness of such paradigms by robotics experts, not least due to a general lack of maturity of the robotics software engineering domain (García et al. 2020). On the other hand, the field of SPLE still assumes a low-tech approach, targeting static and fine-grained variability (Berger et al. 2013, 2014) as opposed to dynamic variability with late binding. For the latter, we believe that the problem lies in the lack of means for managing such features centrally, including techniques for keeping an overview understanding and using that for centralized and controlled configuration. This problem is exacerbated by the variety of mechanisms to implement variability in robotics software; there, various ad hoc mechanisms exist, but no standardized solution on how to do that within ROS. For instance, dynamic features can be realized using parameterization^31^ or loadable ROS plugins.^32^ Static features with binding time compile-time might need a preprocessor and some inclusion into the build system; so, while a diversity of techniques exist, there are no guidelines on which to use and how in robotics—a call to arms for future research.

#### Recommendations

Robotic companies make use of well-known middleware and frameworks to ease software development (Obs. 22). The most common example is ROS, a middleware that provides a framework and enables certain software engineering practices (e.g., modularity, reusability) and enforces development paradigms such as CBSE (García et al. 2020). In this light, mainstream middleware used by roboticists should also integrate means and techniques for planning, designing, and implementing variability. The recommendation is two-fold: (i) mechanisms comprising techniques to model the variability with binding times and modes, and (ii) mechanisms for realizing that variability in the actual robotics source code. The latter should be ideally integrated into concrete technological support by middleware, frameworks, or robotic reference architectures (García et al. 2018, Kramer and Magee 2007), but could also be in the form of guidelines, templates, or design patterns. This would improve current software development practices and also raise the awareness of industrial practitioners. We propose as an action for researchers the study of current middleware and the identification of paradigms that might benefit developers and practitioners. Developers and industrial practitioners could use that information to integrate those paradigms into current mainstream middleware that meets industrial needs. Industrial practitioners might share their experiences to conceive, lead by researchers, guidelines, templates, or design patterns for the systematical plan, design, and implementation of variability. We believe that the community has been taking the right steps in the last years towards adopting systematic approaches to manage variability. Promising examples are the framework extensible via plugins of the official navigation stack for ROS2 (Macenski et al. 2020) and initiatives such as RobMoSys^33^ that are working to apply model-driven methods and tools to current robotics software development processes.

### Variability-Related Challenges

#### Observations

Only few papers from our SLR identify variability-related challenges in industrial applications. This is probably due to the fact that most papers present approaches that have been applied only to research prototypes and not to industrial case studies. However, we saw commonalities between the most significant variability-related challenges proposed in the literature and some of the challenges stated by our interviewees. The commonalities cover challenges concerning managing the combinatorial explosion of product configurations (Obs. 34) and the association of high-level user requirements to individual robot configurations (Obs. 36). The most repeated challenge in the SLR concerns testing (Obs. 33) and certifying (Obs. 23) products and product configurations.

#### Hypotheses

We hypothesize that testing and certification are considered among the most pressing challenges by both the SLR and the interviewees due to the inherent complexity of service robots (Foster et al. 2020), especially those that can be customized. Software product line (SPL) testing, part of SPL certification, is still considered a challenge by the literature due to the variety of products that can be derived from a single product line (Braga et al. 2012; Pérez et al. 2009; Engström and Runeson 2011). This is especially important for cyber-physical systems, which integrate hardware and software components into systems that operate in real-world scenarios. This integration needs to be assessed through system testing. In this light, we hypothesize that a core issue related to this challenge is that variability in complex systems introduces interdependencies among variation points and features. These interdependencies may lead to feature interactions, which are often difficult to identify solely based on the behaviors of the features in isolation. So, bindings among features need to be modeled and tested, to verify the absence of incorrect bindings. Until the features and variation points are bound, complete integration and system testing cannot be performed.

#### Recommendations

According to the literature (Braga et al. 2012), a common approach to validate complex systems is to test each of its components separately. This approach is followed by PAL Robotics, as discussed in Obs. 33. To simplify this approach, robotic companies might strive to reduce redundant testing and to reuse test artifacts so to reduce testing effort. We see the necessity of test automation mechanisms that can ease software development for companies, as previously highlighted by Engström and Runeson (2011). These mechanisms should support developers in performing large numbers of isolated tests (unit tests) and integration tests to validate the interaction and bindings of features. Because service robots operate in real-world scenarios, the mentioned mechanisms should support real-world data over prolonged timeframes to evaluate possible failures and unexpected environmental events. As a recommendation for researchers, we identify a need for testing mechanisms that consider real-world data to perform automated and systematic testing of variant-rich systems. To achieve this goal, collections of real-world and industry-based data and scenarios—which in our opinion should be conducted by industrial practitioners—are required for generation test cases, as mentioned by Cleland-Huang et al. (2018). In this light, automatic test generation (Nebut et al. 2006) could complement the automated testing of variant-rich systems as a cost-efficient and reliable solution, as discussed by Mossige et al. (2014).

Finally, a well-defined SPL simplifies the management of variability of complex systems and eases the definition and reuse of test artifacts (Engström and Runeson 2011). Initiatives such as the research incubator Dronology (Cleland-Huang et al. 2018), if conducted together with industry, could pave the road towards flexible, yet well-tested cyber-physical systems.

## Threats to Validity

We discuss the threats to the validity of our empirical study, using the standard categorization by Wohlin et al. (2012).

### External Validity

According to Maxwell (1992), generalizability is the extent to which we can extend our findings to other situations or cases than the ones we focused on in this case study. It is important however to remark that describing particular cases and deriving insights is the main goal of qualitative research rather than arriving at generalizable conclusions, as discussed by Creswell and Creswell (2017). We might have incurred a sampling bias since we asked for knowledgeable practitioners from both studied companies, who may have a positive attitude toward the topic. Our interviewees work with robotic systems, covering a wide range of domains, contexts, and backgrounds. They come from two different companies whose headquarters are located in two different countries, namely Denmark and Spain. The practices, characteristics, and challenges we describe are applicable to service robots in similar domains.

Minimizing potential threats to the external validity of our study was key for us due to the nature of an SLR, which aims at being as generalizable as possible. As an attempt to mitigate this issue we adopted conservative exclusion criteria that disregarded grey literature papers, position papers, workshop summaries, and short papers.

### Construct Validity

Practitioners in the robotics domain come from a variety of backgrounds and, therefore, might use different terminology (García et al. 2020), which was the main threat to construct validity. To mitigate this threat, we introduced and explained terms that could lead to confusion, including *requirements*, *features*, *variants*, and *mission* during the interviews.

Another potential threat to construct validity is the misinterpretation of our interviews. To avoid this issue, we made recordings and transcribe all of them, so we could have a word-by-word analysis. However, other behavioral aspects from our interviewees such as gestures, pauses, or irony were not captured in our transcripts. This could lead to some misinterpretation of the statements. To alleviate this problem we referred to the recordings to clarify confounding parts in the transcripts.

### Internal Validity

A large number of our interviewees are volunteers, most of whom we did not know personally. According to Wohlin et al. (2012), the selection of volunteers may threaten internal validity because they tend to be more motivated and suited for a new task than the whole population, and therefore they are not representative of the whole population. As discussed by Verner et al. (2009), a reduction of this bias to minimize the effect of confounding factors can be made by increasing the sample of the study, making it more diverse, and increasing the rates of volunteering. With this aim, we (i) asked interviewees about practitioner colleagues who may fit in our selection criteria and might be interested in our study, (ii) recruited practitioners with a variety of roles from both companies, and (iii) strove to show our study as theoretically and practically relevant for our potential interviewees.

According to Creswell and Creswell (2017), the researcher cannot avoid influencing the setting of an interview. The context of the interview, the way questions are phrased and possible reactions from the interviewer might influence the behavior of the interviewee and subsequent answers. Creswell states that even though the interviewee is *always* influenced by the interviewer, there are things one can do to mitigate such bias. We strove to formulate the questions in an unbiased fashion and to identify potentially misleading questions.

### Conclusion Validity

A risk of analyzing qualitative data is the possibility of being biased by the researchers’ background, values, and theories. Also, the analysis of qualitative data through coding as we performed may be subject to the researcher’s interpretation. However, we were not evaluating a solution, method, or tool and therefore we did not risk being biased in trying to arrive at certain conclusions. To mitigate this bias, we conducted the qualitative analysis of our data collaboratively, that is, the open coding was performed by two authors. We also cross-checked and refined the codebook obtained after the analysis iteratively between two authors. Furthermore, we conducted a workshop among all the authors to discuss the codebook to align ideas and concerns and to enhance our study’s validity.

Another potential threat to conclusion validity is given by the substantial work carried out by a single researcher during the conduction of the SLR. The potential bias introduced by this researcher was mitigated by the inclusion of a second researcher during the selection of primary studies and data extraction as quality control. These two researchers held iterative discussions and led an informal workshop with five of the authors of this paper to iteratively refactor the data extraction process.

To make a meaningful evaluation of our findings we discussed them among all authors, including one industrial practitioner. This allowed us to evaluate whether our findings were in line with industry reality.

## Related Work

We already compared specific aspects of our study with related works in the previous sections. Specifically, in Section 4.4 we make a comparison with the literature on drivers of variability, in Section 5.7 with the literature on variability management practices, and in Section 6.4 with the literature on variability-related challenges. In the current section, we complement the comparison with a discussion of empirical studies that focus on variability management in other domains. Overall, we confirm some of the variability drivers, variability management practices, and variability challenges already identified in other domains, and we add characteristics that are specific from robotics. In the following, we provide a more detailed comparison for each of the identified related works.

Berger et al. (2020) study systematic variability management techniques and software product line engineering (SPLE) concepts in twelve cases that include domains such as automotive, aerospace, or railway systems. The authors use a multiple-case study to identify challenges to the adoption of systematic variability management. They claim that hardware is one of the most significant drivers of variability among their studied industrial cases and that the automotive domain is the most advanced in terms of adopting SPLE concepts. The work also identifies some characteristics of the environment driver of variability, however, without putting much focus on inclusion of humans, as, instead, we do. The driver of variability that is instead completely missing is mission. This is not surprising since, currently, mission is a rather specific driver of variability of the robotic domain. However, we expect that it will become increasingly important also for autonomous systems used in other domains. For example, when autonomous vehicles will be deployed in the streets of our cities, we will need to “program” their behavior. Berger et al. (2020) conclude that one of the main challenges of adopting SPLE concepts is tool-integrating problems due to the diversity of tools and artifacts needed for software development in the studied domains. Also for what concerns challenges, in this work we identified challenges that are specific of the robotic domain, e.g., related to the involvement of non-technical operators who might not be skilled in computer science and/or robotics.

Krüger et al. (2017) identify and categorize relevant aspects of variability as well as challenges of variability modeling of cyber-physical systems (CPS). The identification of aspects and challenges of variability stems from their experience. This work does not explicitly identify drivers of variability and variability management practices. For what concerns challenges, the main identified ones are modeling, interaction, configuration, and quality. We are very aligned with their findings for what concerns configuration, which should not be confined into design-time configuration. We did not find much emphasis in modeling but instead we found challenges in integration also intended in the context of iterative development that poses some constraints in upfront modeling. About quality, we indeed found the challenges related to safety. In particular, we found challenges related to reliability of variant-rich systems. Finally, as discussed for the previous work, we identified challenges that are raised by the involvement of end-users.

The study by Flores et al. (2012) details how General Motors^34^ applied SPLE to their organization in the automotive domain. The authors introduce the challenges the company faced to apply the SPLE paradigm to such a big organization, highlighting the complexity levels of variants. The authors complement the study by explaining the technical and organizational lessons learned in an experience report style. Similarly, the study by Dumitrescu et al. (2013) reports on the authors’ experience in modeling a family of parking brake systems and discusses the requirements of Renault regarding variability management. The authors discuss modeling techniques and tools to support variability modeling and present an approach for adopting the product line paradigm in systems engineering in the context of Renault. The paper concludes with the challenges the authors were confronted with when implementing their approach. Thomas et al. (2011) also focus on the implementation of SPLE to the automotive domain. Concretely, the authors discuss the challenges of a hypothetical introduction of a software product line approach to the development of automotive-based applications using the AUTomotive Open System ARchitecture (AUTOSAR), proposed by Fürst et al. (2009). These works do not explicitly identify drivers of variability, as well as variability management practices. About the challenges, it is quite difficult to make a comparison with our work since these papers are mostly reporting some experiences and the challenges are at a different level of granularity since are those that the company faced in applying the SPLE paradigm or in bridging the gap between product line and systems engineering. Examples of these challenges are that vehicles are complex, the organization is very large, huge number of variants, etc.

On a more general note, the study of Chen and Babar (2010) uses focus groups (Kitzinger 1995) to gather data about challenges faced by industrial practitioners in variability management. The authors distinguish between technical and non-technical challenges and discuss their results by comparing them with the study by Bosch et al. (2001). The latter is an experience report where the authors identify and describe variability issues related to variability management of software product lines. As opposed to Chen et al., Bosch et al. discuss their identified challenges relating them to phases of a software product line life cycle. These works do not explicitly discuss drivers of variability and variability management practices. Our work do not focus much on non-technical issues. Moreover, our work is tailored to the robotics domain and reports on challenges that are specific from the domain, like mission specification and user-friendliness coming from the need of involving users without a deep knowledge in computer science and/or robotics.

On the same note, Chen and Babar (2011) conducted a systematic literature review of variability management approaches in software product lines. This work does not explicitly discuss drivers of variability and challenges. Instead, the work collected 91 approaches for dealing with variability management during different development phases. We found some relation with our management strategies and mechanisms.

An interesting conclusion of the work is that the variability management approaches used in those papers were not evaluated using scientifically rigorous methods. Even though we do not investigate this aspect in our work, considering the findings in Bozhinoski et al. (2019), we expect similar results to hold also in robotics. In fact, Bozhinoski et al. (2019) surveys approaches managing safety for mobile robotic systems and concludes that very few of the existing solutions are compliant to standards^35^ that specifically target safety aspects, and also that existing solutions are not yet ready to be used in everyday life.

## Conclusion

We presented an empirical study on variability in service robotics based on the state-of-practice and the state-of-the-art. We conducted (i) a multiple-case study relying on a total of eleven interviews with practitioners from two companies, and (ii) a systematic literature review in which we considered 213 papers and thoroughly analyzed 30 of them. We triangulated from these two sources as well as from another source: our own previous experiences. We contribute: (i) characteristics and impacts of drivers of variability in service robotics, (ii) variability management practices applied by service robotics companies, (iii) challenges faced by industrial practitioners as a result of variability in their products, (iv) a discussion of the gap between state-of-practice and state-of-the art, with formulated hypotheses to explain our observations. Our results contribute to improving the empirical understanding of the specific variability-related characteristics and challenges of the service robotic domain. We hope that these results will support tool builders, practitioners, and researchers to raise awareness for variability, devise better tool support, as well as to guide future research.

Among the findings, we highlight the following four:

- **Abstraction and customizability**: We learned the challenge for practitioners of balancing raising the level of abstraction in their robot control systems and customizing them to specific requirements and scenarios. While higher levels of abstraction could ease variability management and integration, tailored solutions pose great levels of efficiency and effectiveness. Similarly, for robots to operate in several scenarios developers need to both realize variability and integrate robust abstractions. The former represents what can be planned before the deployment or execution of the robotic mission, whereas the latter are meant to deal with unplanned situations that may occur in the scenarios in which robots operate. To better understand what practices and tools lie in any of these two categories, empirically-validated criteria are needed. This is, however, out of the scope of this paper and we consider it valuable future work.
- **Robustness and variability**: Either using variability mechanisms or robust abstractions, robotic applications must be able to operate robustly in a variety of scenarios, many of them being only partially known and controllable. To support the robustness of their systems, our studied companies make use of modeling notations, two important ones being finite-state machines and behavior trees. Behavior trees are found particularly useful for scaling up to scenarios with many sources of variability since they focus on high-level actions that are coordinated in an asynchronous request-reply pattern.
- **Installation process**: In the robotic domain, part of the variability can only be resolved in the installation phase, e.g., properly mapping the operational environment, specifying the missions to be performed and configuring the robots accordingly, defining adaptation rules of the environment. Until now this phase is specifically important for professional robots. In the near future, with the advent of multi-purpose and more complex robots to be used in everyday life, the installation process could be delegated to customers—as nowadays happens with consumer robots such as vacuum cleaning robots—, which, in general, will lack knowledge in robotics and computer science. This will ask for easy way means to install the robots in the customer environment and to correctly specify safe missions robots should perform.
- **Standard interfaces and ecosystem**: The service robotics domain needs mature solutions for managing reusable and modular libraries and packages with standardized and harmonized interfaces. Decoupling between hardware and software is a necessary step. Then, libraries and packages should be organized in an ecosystem where various companies, research institutes, and in general developers can find consolidated and validated solutions and contribute their own. In fact, one of our findings is that the reason behind the success and popularity of ROS might be found in its community and ecosystem.

Table 8 summarizes our actionable results. It lists the characteristics of our identified drivers of variability as well as maps the characteristics to the strategies and mechanisms used by robotics companies. It can be used by researchers and practitioners to match their concerns with actual practices applied by the service robotics industry to tackle those challenges. Table 8 can also serve as a template, to be extended by companies, that may also include their own identified driver characteristics along with the practices applied to deal with the challenges associated with them.

**Table 8:** Mapping of drivers characteristics to management strategies and mechanisms and related challenges

	Driver characteristics	Management strategy	Management mechanism	Related challenges
Environment	Scenario & map layouts	Scenario modeling	Map-editing mechanisms	Conceiving generic solutions
		Installation process	Mechanisms for customers	Parametric configurations
				Performing the installation
	Events	Scenario modeling	Parameters	Conceiving generic solutions
		Generic configurations	Configuration files	Parametric configurations
		Installation process	Adaptation rules	Performing the installation
		Customers’ feedback	Contextual navigation	
			In-house tools	
	Environment	Scenario modeling	Parameters	Conceiving generic solutions
		Generic configurations	Configuration files	Parametric configurations
		Installation process	Map-editing tools	Performing the installation
		Customers’ feedback	Adaptation rules	
			Contextual navigation	
	Inclusion of humans	Scenario modeling	Parameters	Conceiving generic solutions
		Installation process	Configuration files	Parametric configurations
		Customers’ feedback	Adaptation rules	
			Contextual navigation	
Hardware	Services	Reuse resources	Software architectures	Generic solutions
		Collaboration with costumers	Middleware	Reliability of variant-rich systems
		Iterative development	Standards and safety layers	Integration
		Decoupling	Version control	Trade-offs
		Harmonized interfaces	Configuration files & parameters	Influence from environment
		Inter-department communication	Libraries	
		Customizable codebase		
	Capabilities	Reuse resources	Software architectures	Generic solutions
		Collaboration with costumers	Middleware	Hardware-driven assumptions
		Iterative development	ROS_control	Reliability of variant-rich systems
		Decoupling	Standards and safety layers	Integration
		Harmonized interfaces	Version control	Trade-offs
		Inter-department communication	Configuration files & parameters	Handling real-time components
		Customizable codebase	In-house tools	Influence from environment
			Libraries	
	Embodiment	Iterative development	Software architectures	Hardware-driven assumptions
		Inter-department communication	Middleware	Multiple options for hardware
		Add-ons	Standards and certifications	Lack of standardized hardware
				Integration
				Aligning hardware conventions
				Trade-offs
				Influence from environment
	Customer requirements	Reuse resources	Software architectures	Reliability of variant-rich systems
		Collaboration with customers	Middleware	Lack of standardized hardware
		Iterative development	ROS control	Integration
		Decoupling	Standards and safety layers	Trade-offs
		Harmonized interfaces	Version control	Influence from environment
		Inter-department communication	Configuration files & parameters	
		Customizable codebase	In-house tools	
		Add-ons	Libraries	
Mission	Operator’s expertise	Generic missions	Finite-state machines	Mission specification
			Task frameworks	Promoting user-friendliness
			Behavior trees	
			GUIs	
			Navigation frameworks	
	Human-robot interaction	Generic missions	Finite-state machines	Promoting user-friendliness
			Task frameworks	
			GUIs	
	Events	Generic missions	Finite-state machines	Mission specification
		Fail safely	Flowcharts	Promoting user-friendliness
			Behavior trees	
			Navigation frameworks	

Our results give rise to the following future work:

- Consolidate the drivers of variability in service robotics in a common ontology, acting as a standard.
- Develop better mechanisms for realizing variability in the actual robot code.
- Define guidelines, templates, and design patterns for planning, designing, and implementing variability.
- Develop testing techniques that consider real-world data to perform automated testing of variant-rich systems by systematically enforcing reducing redundant testing and reusing test artifacts.
- Define a variability-aware development process that builds on current practices in both academia (independent open-source community efforts) and industry (in-house hardware product lines).

## Data Availability

The interviews’ codebook and interview guide as well as the SLR’s protocol, data extraction templates, results, and search strings that support the findings of this study are available in figshare with the identifier http://doi.org/10.6084/m9.figshare.13650362. A comprehensive description of the data is available on a dedicated website https://sites.google.com/view/variability-robotics/home.

## Footnotes

1 https://www.iso.org/standard/55890.html

2 https://www.marketsandmarkets.com/Market-Reports/ivd-bric-market-198.html

3 https://www.forbes.com/sites/richblake1/2020/04/17/in-covid-19-fight-robots-report-for-disinfection-duty

4 https://www.mvpromedia.com/article/robots-to-assist-fight-against-covid-19-in-hospitals/

5 https://pal-robotics.com/robots/tiago

6 https://www.youtube.com/watch?v=wzQoWtEHbKA

7 http://www.co4robots.eu

8 https://www.bosch-ai.com

9 https://www.qsrinternational.com/nvivo-qualitative-data-analysis-software/home

10 https://eu-robotics.net/sparc/upload/about/files/H2020-Robotics-Multi-Annual-Roadmap-ICT-2016.pdf

11 https://www.iso.org/standard/70939.html

12 https://www.youtube.com/watch?v=eN9Dl1zG3no

13 https://www.gobe-robots.com

14 http://www.uvd-robots.com

15 http://ptr-robots.com

16 http://pal-robotics.com/wp-content/uploads/2020/05/TIAGo-Datasheet.pdf

17 https://www.youtube.com/watch?v=GdMmyrzIP8o

18 https://wiki.ros.org/rqt_reconfigure

19 https://yaml.org

20 http://wiki.ros.org/roslaunch

21 http://wiki.ros.org/rviz

22 http://wiki.ros.org/smach

23 https://www.scaledagileframework.com

24 http://blog.pal-robotics.com/tiago-bi-manual-robot-research/

25 http://wiki.ros.org/Distributions

26 https://wiki.ros.org/ros_control

27 https://github.com/ros/rosdistro

28 https://behaviortree.github.io/BehaviorTree.CPP

29 https://github.com/splintered-reality/py_trees

30 http://wiki.ros.org/move_base

31 http://wiki.ros.org/Parameter%20Server

32 http://wiki.ros.org/pluginlib

33 https://robmosys.eu

34 https://www.gm.com

35 Like IEC61508—Functional Safety of Electrical/Electronic/Programmable Electronic Safety-related Systems, ISO13855—Safety of machinery—Positioning of safeguards with respect to the approach speeds of parts of the human body, ISO13482—Robots and robotic devices—Safety requirements for personal care robots
